# The spatial distribution of diagnosed Type 2 diabetes mellitus and cardiovascular disease incidence in Valencia, 2015–2022: a retrospective, registry-based study

**DOI:** 10.1186/s12889-026-27160-3

**Published:** 2026-03-29

**Authors:** Alfonso Gallego-Valadés, Tamara Alhambra-Borrás, Antonio López-Quílez, Celia Bañuls, Jorge Garcés-Ferrer, Estrella Durá-Ferrandis

**Affiliations:** 1 University of Valencia, Polibienestar Research Institute, Valencia, Spain; 2 Department of Statistics and Operational Research, University of Valencia, Valencia, Spain; 3 Fundación para el Fomento de la Investigación Sanitaria y Biomédica de la Comunitat Valenciana, Translational Research in Nutrition and Metabolism Group, Valencia, Spain

**Keywords:** Spatial distribution, Spatial epidemiology, Non-Communicable Diseases (NCDs), Urban Health, Type 2 diabetes mellitus (T2DM), Cardiovascular disease (CVD), Socioeconomic inequalities

## Abstract

**Background:**

Cardiovascular disease (CVD) and type 2 diabetes mellitus (T2DM) are major contributors to global morbidity and mortality, shaped by social, environmental and behavioural determinants. Assessing the spatial distribution and socio-economic stratification of these conditions at fine geographic scales can inform place-based public health strategies. This study characterises the spatial patterns, socioeconomic gradients and temporal evolution of diagnosed CVD and T2DM incidence in the urban context of Valencia, Spain, for adults aged ≥ 40 years from 2015 to 2022.

**Methods:**

We conducted a retrospective, registry-based, open cohort study using routinely collected clinical data linked to population registries across 26 postcodes in Valencia. Incident CVD and T2DM cases were identified using prespecified ICD-9/10-CM codes. Person-years at risk were aggregated annually by census tract, sex and age. Age- and sex-adjusted incidence rates were derived by direct standardisation. Spatial clustering was evaluated using global Moran’s *I* with first-order queen contiguity weights. Socioeconomic gradients were assessed via tract-level mean income per unit of consumption, both categorically (quartiles) and continuously using generalised additive models to analyse non-linear associations.

**Results:**

Over 3.4 million person-years, we identified 19,902 first CVD events and 27,245 T2DM diagnoses, corresponding to crude incidence rates of 5.85 and 8.55 per 1,000 person-years, respectively. Age- and sex-adjusted incidence exhibited inverse gradients across income quartiles for both outcomes, steeper for T2DM. Spatial autocorrelation was significant for all incidence surfaces (*p* < 0.001), markedly stronger for T2DM (*I* = 0.462) than for CVD (*I* = 0.174). Non-linear modelling revealed pronounced socioeconomic gradients at lower income levels, particularly for T2DM.

**Conclusions:**

In Valencia, diagnosed CVD and T2DM incidence show clear socioeconomic and spatial inequalities, with pronounced clustering and steep income-related gradients for T2DM. These results underscore the importance of geographically targeted interventions to mitigate cardiometabolic health disparities.

**Supplementary Information:**

The online version contains supplementary material available at 10.1186/s12889-026-27160-3.

## Background

The global burden of chronic diseases has increased substantially in recent decades, driven by the interaction of social and environmental determinants of health. Cardiovascular disease (CVD) and type 2 diabetes mellitus (T2DM) are shaped by broader structural conditions, including population ageing, urbanization, ethnic migration, and persistent poverty [1–3]. Environmental features such as the built environment, access to green spaces, and neighbourhood socioeconomic conditions influence opportunities for healthy living, while behavioural factors including diet, physical activity, alcohol consumption, and tobacco use remain major modifiable determinants of cardiometabolic risk [1, 4].

Both conditions are expected to remain major public health challenges in the coming decades. Chong et al. [5] projected that the global crude prevalence of CVD will increase from 598 million cases in 2025 to 1.14 billion in 2050, while CVD-related deaths will rise from 20.5 million to 35.6 million. The World Health Organization [6] estimates that CVDs account for around 32% of all global deaths, with heart attacks and strokes causing approximately 85% of these fatalities. Diabetes shows a similar trajectory. A systematic analysis estimated that 529 million people were living with diabetes worldwide in 2021, with T2DM representing 96.0% of all cases [7]. More recent evidence indicates that diabetes affected 11.1% of the global adult population in 2024 and may reach 13% among adults aged 20–79 years by 2050 [8].

In Spain, a nationwide population-based cohort study [9] reported an age- and sex-adjusted diabetes incidence of 11.6 cases per 1,000 person-years and an incidence of known diabetes of 3.7 cases per 1,000 person-years. Bennett et al. [10] found that T2DM incidence declined between 2009 and 2018 in the 55–69 and ≥ 70 age groups in both sexes, particularly among older adults, while increasing slightly in adults aged 40–54 years. By contrast, CVD incidence rose across most age groups and in both sexes during the same period, with the largest increases observed among individuals aged 40–54 years [10].

### Socioeconomic gradients in cardiometabolic disease burden

Although CVD and T2DM incidence has stabilised or declined in many high-income countries, cardiometabolic diseases continue to increase in low- and middle-income settings [11]. In Spain, lower socioeconomic status (SES) is associated with a higher burden of cardiometabolic risk factors and disease [12, 13]. Using data from the 2017 Spanish National Health Survey, Gullón et al. [12] identified clear socioeconomic inequalities in diabetes, hypertension, obesity, smoking, and high cholesterol, with lower social classes consistently showing higher prevalence. These inequalities were generally stronger among women for diabetes, hypertension, and obesity, and regional analyses showed marked heterogeneity, with the widest diabetes inequalities observed in the Comunitat Valenciana and Asturias.

Bilal et al. [13], in a study of 269,942 adults aged ≥ 40 years in northeastern Madrid, likewise found that lower neighbourhood SES was associated with higher diabetes prevalence, poorer glycaemic control, and greater incidence, with steeper gradients among women. Whereas Gullón et al. [12] highlighted regional variability, Bilal et al. [13] described a more uniform gradient within a single urban setting. Together, these studies suggest that socioeconomic disadvantage is a robust predictor of diabetes burden, although the magnitude and expression of these inequalities vary across contexts. This supports the need for further research on the spatial distribution of diagnosed T2DM and CVD incidence.

### The spatial epidemiology of NCDs

Spatial analysis is central to identifying geographical patterns in disease incidence and prevalence and to examining area-level associations between health outcomes and contextual determinants. In the field of NCDs, including CVD and T2DM, several studies have assessed the spatial overlap between disease burden and the distribution of socioeconomic, behavioural, and demographic drivers.

Spatial clustering is a key feature of spatial patterning [14], and spatial autocorrelation measures the extent to which values in neighbouring areas are correlated. Moran’s *I* is the most widely used global indicator of spatial autocorrelation [15–17]. Examples include Gómez-Peralta et al. [18], who identified spatial clustering of hypoglycaemic events in Andalusia; Wu et al. [19], who found strong positive spatial autocorrelation in T2DM incidence in New South Wales, Australia; and Zhang et al. [20], who reported similar findings in Zhejiang Province, China. The spatial distribution of CVD and T2DM partly reflects the spatial distribution of their determinants. Wu et al. [19] reported positive correlations between T2DM incidence and both obesity prevalence and insufficient physical activity, while Zhang et al. [20] suggested a positive association between neighbourhood deprivation and T2DM incidence. However, analyses based on crude rates may be confounded by the demographic composition of areas, so age- and sex-adjusted rates are important to reduce this bias.

Most previous studies have used territorial units larger than the Spanish census tract, whereas fine-grained analyses remain less common. This is relevant because conclusions about spatial patterning may vary with the scale of analysis, a problem known as the Modifiable Areal Unit Problem (MAUP) [21]. As area size increases, so does internal heterogeneity in socioeconomic conditions, environmental exposures, and risk profiles, weakening the correspondence between aggregated area measures and individual experience. Some studies, such as Jordan et al. [22], have used address-level georeferenced data and local cluster analysis with the Getis-Ord Gi* statistic [23, 24], showing that significant clusters of predicted risk and observed CVD incidence can be identified at a finer spatial scale.

### Objective

The aim of this study is to analyse the spatial distribution of diagnosed T2DM and CVD incidence in the city of Valencia (Spain) between 2015 and 2022 using population-based Electronic Health Records (EHR) data, in order to identify spatial patterns, areas with higher cardiometabolic burden, and their area-based association with SES. More specifically, the study seeks to generate evidence to inform targeted public health interventions and resource allocation, particularly in socioeconomically disadvantaged areas where cardiometabolic burden is higher.

## Methods

### Study design and setting

We conducted a retrospective, registry-based, open cohort study using routinely collected clinical data (RCCD) from EHRs in Valencia (Spain). The study area covered 26 postcodes within the municipality of Valencia, and the population was restricted to adults aged ≥ 40 years between 2015 and 2022. This threshold was adopted as a pragmatic design restriction to reduce potential misclassification of incident onset in an open cohort defined by annual residence within the study area, since residential mobility is concentrated in young adulthood and declines thereafter [25]; excluding younger adults therefore reduces the inclusion of the most mobile age groups. Full definitions of the spatial-temporal boundaries of the study and considerations regarding the observation framework are provided in the Supplementary Material.

### Data sources

Individual-level demographic and residential information was obtained from the regional population register (SIP), which provides stable information on age and sex and yearly updates on the census tract of residence. Morbidity data were obtained from the outpatient EHRs archive (SIA-GAIA), coded in both ICD-9-CM and ICD-10-CM. Datasets were deterministically linked via a pseudonymised unique identifier. Before linkage, longitudinal internal consistency checks were applied, and known low-level inconsistencies in sex or year of birth were resolved using modal imputation. Additional details on data provenance, linkage, quality assessments and regulatory safeguards are presented in the Supplementary Material.

### Study population and follow-up

An open, retrospective cohort was constructed by generating one record per individual and calendar year in which the person was resident in the study area. Person-time accrued exclusively in discrete years with observed residency, and residence was reassessed annually to reflect moves into or out of the study area. Implausible or missing demographic fields resulted in exclusion. Person-years were cross-classified by five-year age group, sex and census tract. The cohort structure, internal checks and inclusion-exclusion rules are documented in the Supplementary Material.

### Outcome definition and incidence estimation

CVD and T2DM onsets were identified using prespecified ICD code sets. A two-tiered coding strategy distinguished (i) a comprehensive screening set and (ii) a subset of codes clinically compatible with an initial or acute presentation (“incident-compatible”). Individuals with any known disease-specific diagnosis recorded in years when they were outside the spatial-temporal boundaries of the study were classified as prevalent at the first subsequent year of (re-)entry, including this one (inclusive cut-off). Similarly, if the earliest secondary or complication-type code occurred strictly before the earliest incident-compatible code, the individual was treated as prevalent from that calendar year onwards. Among the remaining at-risk population, incident cases were assigned to the earliest known incident-compatible diagnosis occurring between 2015 and 2022 while under observation, and person-time was censored at the onset year. Annual numerators and denominators were then aggregated across sex-age strata by census tract and year for area-level rate estimation. Operational rules, full coding logic, tie-resolution and a subject-level flowchart of the workflow are provided in the Supplementary Material.

### Small-area units, areal harmonisation and socioeconomic indicator

Census tracts constituted the spatial unit of analysis. Because tract boundaries were periodically revised over 2015–2022, all population-at-risk denominators and incident counts were first expressed in their original annual geometries and subsequently projected onto the 2022 census tract layout using area-weighted geometric interpolation constrained by a built-up land mask. This harmonisation ensured full mass preservation and eliminated artefacts introduced by tract subdivision or reconfiguration, yielding a consistent spatial framework for all downstream analyses. Census tract-level SES was measured through the mean income per unit of consumption from the *Atlas de Distribución de Renta de los Hogares* (National Statistics Institute). This variable was operationalised in two forms: (i) as a continuous tract-level measure using 2022 values for cross-sectional analysis; and (ii) as year-specific income quartiles for stratified incidence estimation. Detailed procedures for boundary harmonisation, dasymetric masking and construction of income strata are provided in the Supplementary Material.

### Statistical analysis

#### Crude incidence

Crude incidence rates were calculated as the number of first recorded incident events divided by the corresponding person-years at risk, expressed per 1,000 person-years. Age- and sex-specific crude rates were computed by five-year age bands and sex. Frequencies and proportions of the most common diagnostic entities (ICD-9/10-CM code categories) were summarised separately for CVD and T2DM.

#### Socioeconomic gradients in age-adjusted incidence

Socioeconomic inequalities were assessed using census tract-level mean income per unit of consumption. For stratified incidence analyses, census tracts were ranked within each calendar year according to this indicator and classified into year-specific quartiles (Q1-Q4), labelled as low, medium-low, medium-high, and high income. Because quartile cut points were recalculated separately for each year, the exact euro thresholds vary over time. Age-adjusted incidence rates were then estimated separately by sex and income quartile using direct standardisation. The reference for standardisation was defined as the age distribution of the analytic population with non-missing income information, pooling all sexes and income strata over the period 2015–2022. Adjusted incidence rates were expressed per 1,000 person-years. Gamma confidence intervals for standardised rates were computed using the Fay-Feuer method as described in [26] (see technical details in Supplementary Materials). Person-time contributed and any incident onsets occurring while residing in census tracts with missing income values were excluded from these analyses.

#### Temporal evolution of age-adjusted incidence

Annual incidence series were constructed for men, women, and the total population. For each calendar year, age-adjusted incidence rates were derived by direct standardisation to the 2022 study area-wide age distribution of the population at risk.

Sex-specific and total age-adjusted rates with 95% confidence intervals were estimated for both CVD and T2DM, enabling comparison of temporal trends and sex differentials across the study period.

#### Spatial distribution of age- and sex-adjusted incidence

All annual census tract-level population denominators and incident case counts stratified by age and sex were harmonised to the 2022 census tract geometry. This was achieved using an area-weighted geometric interpolation procedure after restricting all geometries to the study’s urbanised land mask. The procedure preserves totals up to rounding error and allows aggregation of data across years under a common spatial support. Cumulative incidence over 2015–2022 was then calculated at census tract level and jointly adjusted for age and sex by direct standardisation to the combined age-sex distribution of the overall analytic study population. Fully age- and sex-adjusted incidence surfaces were produced separately for adults aged ≥ 40 and ≥ 60 years (aggregated data are available in Supplementary Materials). The ≥ 40 population was treated as the primary population of interest, whereas the ≥ 60 population was examined in a restricted older adult analysis to assess whether the observed socioeconomic gradients and spatial clustering persisted in a population segment with a higher absolute cardiometabolic burden. Because the ≥ 60 population is nested within the ≥ 40 population, these thresholds were used as complementary age-based restrictions rather than as mutually exclusive age groups.

Global spatial autocorrelation of the age- and sex-adjusted census tract-level incidence surfaces was assessed using Moran’s *I* with first-order queen contiguity spatial weights. Analyses were conducted separately for CVD and T2DM and for the ≥ 40 and ≥ 60 years age groups.

Statistical significance was evaluated using Monte Carlo permutation tests (see technical details and reproducibility in Supplementary Materials).

#### Non-linear associations between area-level income and incidence

To analyse the shape of the association between neighbourhood SES and cardiometabolic incidence, we fitted separate univariable Generalised Additive Models (GAMs) for each outcome (CVD and T2DM) and age group (≥ 40 and ≥ 60 years) at the census tract level. In all models, the exposure was census tract mean income per unit of consumption in 2022, treated as a continuous variable, and the outcome was the corresponding age- and sex-adjusted cumulative incidence rate (per 1,000 person-years) over 2015–2022.

Non-linear relationships were modelled using penalised smooth functions, with the degree of smoothness selected by data-driven criteria. Incidence rates were analysed as continuous outcomes assuming Gaussian errors with an identity link. Model fit and non-linearity were evaluated using approximate F-tests and the effective degrees of freedom (edf) of the smooth term. From each model, predicted incidence rates and 95% pointwise confidence intervals were obtained across the observed income range. To provide an interpretable summary of non-linear socioeconomic gradients, we additionally calculated contrasts in predicted incidence (Δ) over predefined segments of the income distribution. Δ was defined as the absolute difference in model-predicted incidence rates (per 1,000 person-years) between two income values separated by €10,000, evaluated at the lower and upper tails of the income distribution. All modelling steps and graphical outputs are documented in the Supplementary Materials.

## Results

### Cohort composition, follow-up time, and crude incidence

From 2015 to 2022, residents aged ≥ 40 years contributed 3,401,388 person-years of observation free of known prevalent CVD, during which 19,902 first CVD events were identified, yielding a crude incidence rate of 5.85 per 1,000 person-years. In the same age range and period, 27,245 first T2DM diagnoses occurred over 3,187,938 person-years at risk, corresponding to a crude incidence of 8.55 per 1,000 person-years.

Age-specific incidence increased steeply with age for CVD in both sexes (Table 1). Among men, rates rose from 1.21 per 1,000 person-years at ages 40–44 to 24.52 at 85–89 years, remaining similarly high at ≥ 90 years (23.98 per 1,000 person-years). Among women, the corresponding figures increased from 0.57 to 17.48 per 1,000 person-years between the 40-44- and 85-89-year bands, also remaining high in the oldest age group (18.04 per 1,000 person-years). For T2DM, age-specific incidence also rose markedly with age up to late older adulthood, from 2.79 to 18.12 per 1,000 person-years among men (40–44 vs. 70–74 years) and from 1.95 to 13.29 among women (40–44 vs. 75–79 years), followed by a decline in the oldest age groups. Across all age strata and for both conditions, men consistently exhibited higher crude incidence than women (Table 1).

**Table 1:** Crude incidence by age-sex strata (≥ 40 years) in the study area, 2015–2022

	CVD					
	Person-years at risk		Cumulative incidence		Rate per 1000	
Age	Men	Women	Men	Women	Men	Women
40 to 44	255,988	254,503	311	145	1.21	0.57
45 to 49	249,972	253,112	614	252	2.46	1.00
50 to 54	230,238	247,350	982	391	4.27	1.58
55 to 59	200,833	232,018	1,246	533	6.20	2.30
60 to 64	164,463	205,208	1,432	712	8.71	3.47
65 to 69	135,573	183,206	1,482	994	10.93	5.43
70 to 74	112,224	162,802	1,450	1,135	12.92	6.97
75 to 79	80,319	129,040	1,369	1,313	17.04	10.18
80 to 84	53,499	98,854	1,150	1,413	21.50	14.29
85 to 89	29,937	68,433	734	1,196	24.52	17.48
≥ 90	13,013	40,803	312	736	23.98	18.04
	T2DM					
	Person-years at risk		Cumulative incidence		Rate per 1000	
Age	Men	Women	Men	Women	Men	Women
40 to 44	253,047	251,659	707	490	2.79	1.95
45 to 49	244,659	249,068	1130	802	4.62	3.22
50 to 54	221,444	240,668	1,714	1217	7.74	5.06
55 to 59	187,514	221,363	2,114	1615	11.27	7.30
60 to 64	147,746	190,963	2,195	1790	14.86	9.37
65 to 69	118,555	165,117	1,973	1,932	16.64	11.70
70 to 74	96,199	141,699	1,743	1,787	18.12	12.61
75 to 79	69,369	110,786	1,159	1,472	16.71	13.29
80 to 84	48,300	85,488	750	1,103	15.53	12.90
85 to 89	28,753	61,676	417	680	14.50	11.03
≥ 90	13,772	40,093	132	323	9.58	8.06

Table 2 summarises the diagnostic composition of incident CVD and T2DM cases. Cerebral infarction accounted for 5,722 events (28.8%), followed by angina pectoris (3,184; 16.0%), chronic ischaemic heart disease (2,845; 14.3%) and acute myocardial infarction (2,584; 13.0%). Together, these four categories represented approximately 72% of all first CVD diagnoses, with the remaining events distributed across other cerebrovascular entities and miscellaneous CVD codes. For T2DM, incident diagnoses overwhelmingly corresponded to uncomplicated type 2 diabetes without recorded chronic complications (25,763 events; 94.6%), with a smaller proportion coded as type 2 diabetes with hyperglycaemia (1,218; 4.5%) and a residual group of other specified forms (264; 1.0%). The full distribution of diagnostic categories is presented in Table 2.

**Table 2:** Most frequent incident diagnoses (ICD-9/10-CM codes; ≥40 years), 2015–2022

CVD			
ICD-9/10-CM code	Description	n	%
I63	Cerebral infarction	5,722	28.75
I20	Angina pectoris	3,184	15.99
I25	Chronic ischemic heart disease	2,845	14.30
I21	Acute myocardial infarction	2,584	12.98
I67	Other incident-compatible cerebrovascular diagnoses	817	4.11
-	Other incident-compatible CVD diagnoses (miscellaneous)	4,750	23.87
-	Total	19,902	100.00
T2DM			
ICD-9/10-CM code	Description	n	%
250.00/E11.9	Type 2 diabetes mellitus without complications	25,763	94.56
E11.65	Type 2 diabetes mellitus with hyperglycemia	1,218	4.47
-	Other incident-compatible T2DM diagnoses	264	0.97
-	Total	27,245	100.00

### Socio-economic gradients

Age-adjusted incidence rates exhibited clear census tract-level socioeconomic gradients for both T2DM and CVD (Table 3). For T2DM, incidence increased monotonically from the most affluent to the least affluent quartile in both sexes. Among men, age-adjusted incidence ranged from 8 per 1,000 person-years (95% CI 7.71–8.30) in the highest income quartile to 12.8 (12.34–13.17) in the lowest, with intermediate values of 10 (9.66–10.32) and 10.8 (10.48–11.21) in the medium-high and medium-low quartiles, respectively. Among women, rates rose from 5.1 (4.86–5.25) to 9.6 (9.24–9.86), with 6.9 (6.69–7.16) and 7.8 (7.52–8.05) in the intermediate quartiles.

For CVD, inverse income gradients were also observed, although the between-quartile differences were smaller than those seen for T2DM. Among men, age-adjusted incidence ranged from 7.3 per 1,000 person-years (7.01–7.59) in the highest income quartile to 9.1 (8.75–9.44) in the lowest, with corresponding values of 7.9 (7.62–8.21) and 8.1 (7.75–8.37) in the medium-high and medium-low quartiles. Among women, rates increased from 3.7 (3.58–3.91) to 4.9 (4.68–5.09) across the same gradient, with intermediate estimates of 4.1 (3.93–4.27) and 4.4 (4.21–4.58). Across both conditions, men consistently exhibited higher age-adjusted incidence than women at every income level.

**Table 3:** Age-adjusted incidence by census tract SES and sex (≥ 40 years), 2015–2022

Mean income per UC in the census tract of residence	CVD					
	Person-years at risk		Cumulative incidence		Age-adjusted rate per 1,000 *p*-ys.	
Men	Men	Women	Men	Women	Men	Women
Low	355,557	418,938	2,841	2,259	9.09 [8.75, 9.44]	4.88 [4.68, 5.09]
Medium-Low	372,534	456,702	2,698	2,197	8.05 [7.75, 8.37]	4.39 [4.21, 4.58]
Medium-High	405,346	503,391	2,898	2,256	7.91 [7.62, 8.21]	4.10 [3.93, 4.27]
High	376,992	479,032	2,533	2,018	7.30 [7.01, 7.59]	3.74 [3.58, 3.91]
Mean income per UC in the census tract of residence	T2DM					
	Person-years at risk		Cumulative incidence		Age-adjusted rate per 1000	
	Men	Women	Men	Women	Men	Women
Low	326,093	379,373	3,792	3,647	12.75 [12.34, 13.17]	9.55 [9.24, 9.86]
Medium-Low	345,376	424,405	3,542	3,420	10.84 [10.48, 11.21]	7.78 [7.52, 8.05]
Medium-High	378,791	474,245	3,656	3,458	9.99 [9.66, 10.32]	6.92 [6.69, 7.16]
High	364,256	464,373	2,862	2,540	8.00 [7.71, 8.30]	5.05 [4.86, 5.25]

CVD. Total cumulative incidence: 19,700 cases; total population at-risk: 3,368,492

T2DM. Total cumulative incidence: 26,917 cases; total population at-risk: 3,156,912

Some cases and persons at-risk have been lost due to missing income values for the classification of census tracts of residence. Although there may exist some systematic bias in the absence of these values, their impact on the distribution of cases and population-at-risk across strata is likely negligible

### Temporal evolution of age-adjusted incidence

**Fig. 1:**
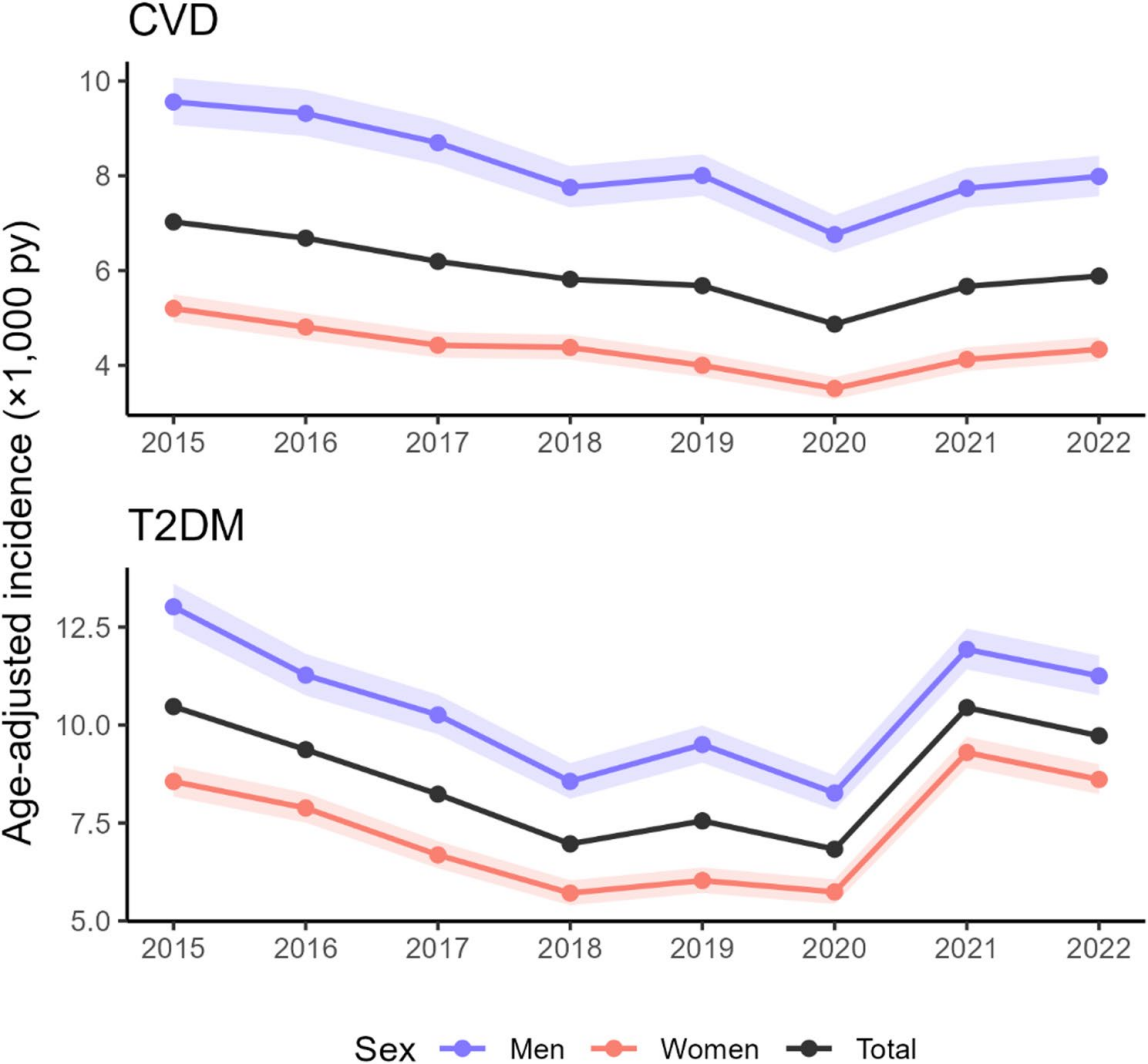
Age-adjusted annual incidence by sex (2015–2022). Upper panel: CVD; lower panel: T2DM

Annual age-adjusted incidence series were derived using direct standardisation to the 2022 city-wide age distribution of the at-risk population (Fig. 1).

For CVD, age-adjusted incidence in the total population declined steadily from 7.03 per 1,000 person-years in 2015 (95% CI 6.77–7.29) to 4.87 (4.66–5.08) in 2020, representing a relative reduction of approximately 31%. Thereafter, rates increased modestly, reaching 5.88 (5.66–6.12) in 2022. Throughout the period, men exhibited substantially higher incidence than women: in 2015, rates were 9.56 (9.08–10.07) per 1,000 person-years in men versus 5.20 (4.92–5.50) in women. In 2022, they remained elevated at 7.99 (7.57–8.42) in men compared with 4.34 (4.09–4.60) in women. The male-to-female incidence ratio remained relatively stable over time, generally between 1.8 and 2.

T2DM displayed a broadly similar temporal pattern at higher absolute levels and with a more pronounced late-period rebound. In the total population, age-adjusted incidence decreased from 10.47 per 1,000 person-years in 2015 (10.14–10.81) to 6.83 (6.58–7.09) in 2020 (a reduction of ≈ 35%), then rose to 10.44 (10.13–10.76) in 2021 and 9.73 (9.43–10.03) in 2022. Men consistently had higher incidence than women (e.g. 13.01 vs. 8.56 per 1,000 person-years in 2015; 11.25 vs. 8.61 in 2022), although the male-female gap was smaller than for CVD, with men rates typically around 1.3–1.6 times those of women.

Overall, both outcomes showed declining age-adjusted incidence up to 2020, followed by a partial rebound in 2021–2022.

### Spatial distribution of incidence

**Fig. 2:**
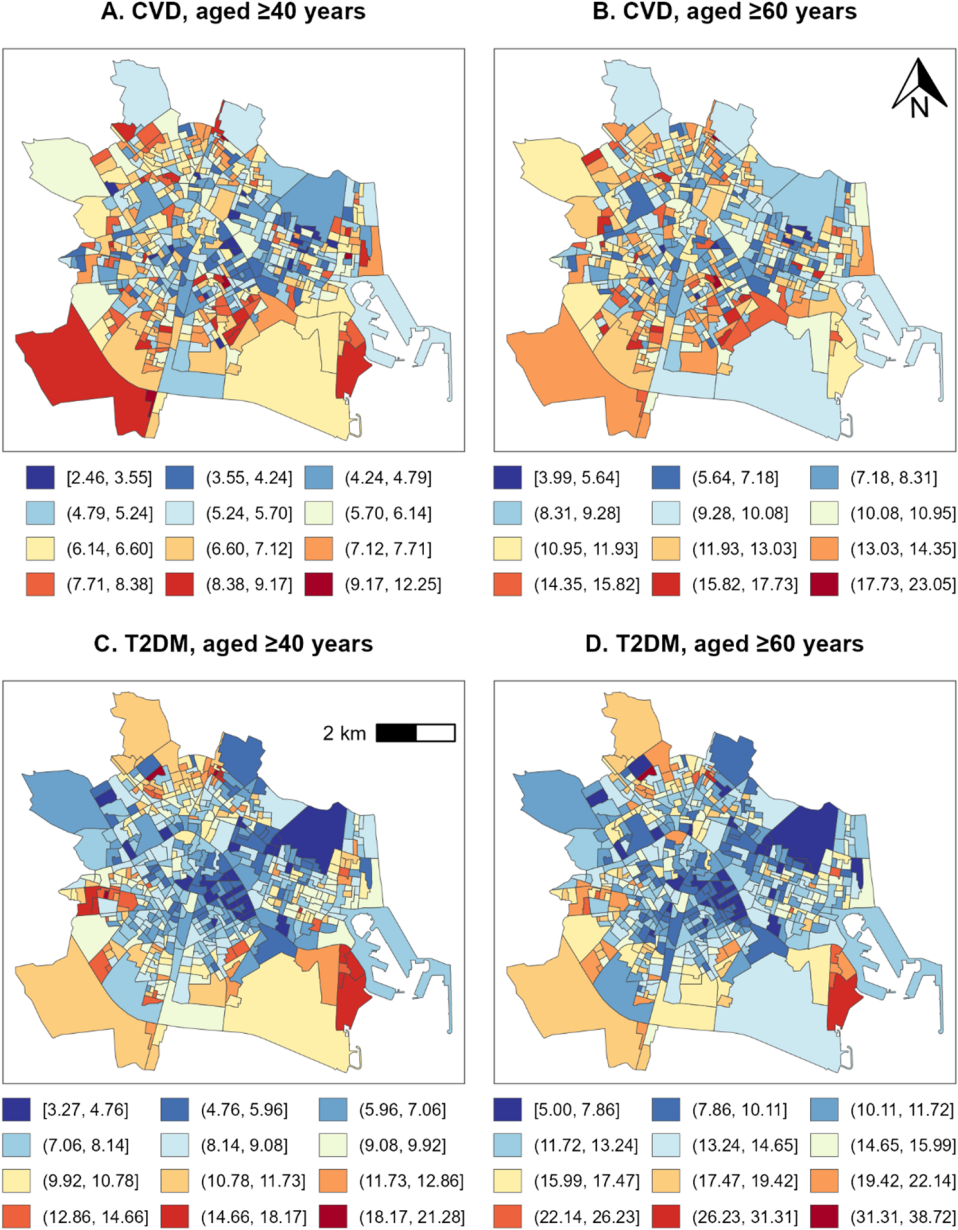
Natural breaks maps (Jenks) of age- and sex-adjusted CVD and T2DM incidence rates^1^

Figure 2 displays the spatial distribution of the age- and sex-adjusted known incidence of CVD (top) and T2DM (bottom), for population aged ≥ 40 years (left) and ≥ 60 years (right), over the period 2015–2022, enabling a comparison of their geographical structure. The maps reveal marked between-neighbourhood variation, with areas of consistently elevated incidence for both conditions forming recognisable spatial clusters – particularly in the case of T2DM. These patterns motivated a formal assessment of spatial autocorrelation.

Spatial autocorrelation was thus assessed using Moran’s *I* set to first order queen contiguity weights, with 999 permutation replicates under a pseudo-randomisation framework for hypothesis testing. All four incidence surfaces, defined by outcome (CVD or T2DM) and age threshold (≥ 40 or ≥ 60 years), exhibited statistically significant positive spatial autocorrelation (*p* < 0.001). For CVD, Moran’s *I* was 0.174 for adults aged ≥ 40 years and 0.107 for adults aged ≥ 60 years, indicating positive spatial autocorrelation in both incidence surfaces. For T2DM, the corresponding values were 0.462 and 0.362. Thus, Moran’s *I* values were higher for T2DM than for CVD in both age-threshold analyses. These results can be verified using the census tract-level data and code chunk described in the Supplementary Materials.

#### Non-linear associations between area-level incidence and income

**Fig. 3:**
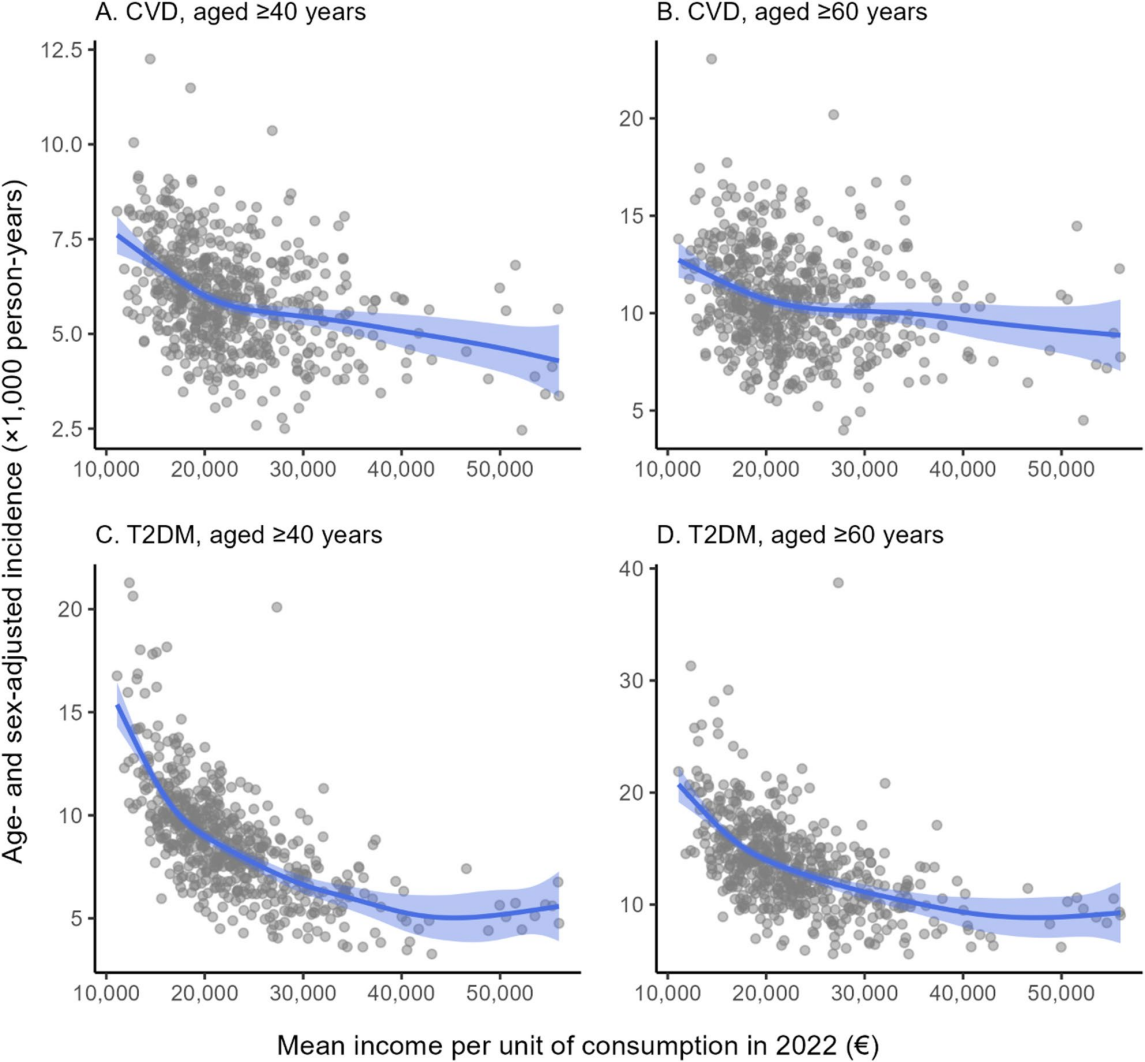
Non-linear associations between census tract-level mean income and adjusted CVD and T2DM incidence^2^

Figure 3 shows the estimated non-linear association between census tract mean income per unit of consumption in 2022 and age- and sex-adjusted incidence, separately for each outcome and age-threshold analysis. Predicted incidence contrasts over €10,000 increments at the lower and upper tails of the income distribution are reported below to facilitate interpretation of the fitted curves. All results presented in this subsection can be replicated using the Supplementary Materials.

For CVD, the fitted association was consistently inverse in both age-threshold analyses. Among adults aged ≥ 40 years, the predicted CVD incidence declined from 7.61 to 5.88 per 1,000 person-years when mean income increased from €11,096 to €21,096, a reduction of 1.73 cases per 1,000 person-years. At the upper end of the distribution, the corresponding decline was smaller, from 4.82 to 4.29 per 1,000 person-years when mean income rose from €45,987 to €55,987 (Δ = −0.53). Patterns were similar in the ≥ 60-year group, with the predicted rate falling from 12.7 to 10.5 per 1,000 person-years (Δ = −2.19) between €11,096 and €21,096, and from 9.33 to 8.86 per 1,000 person-years (Δ = −0.47) between €45,987 and €55,987. These findings indicate a steeper socio-economic gradient at lower income levels and a flatter slope in more affluent areas.

For T2DM, the socio-economic gradient at the lower end of the income distribution was more pronounced. In the ≥ 40-year group, the predicted T2DM incidence dropped from 15.4 to 8.68 per 1,000 person-years when mean income increased from €11,096 to €21,096 (Δ = −6.69), and from 20.7 to 13.6 per 1,000 person-years in the ≥ 60-year group over the same income range (Δ = −7.16). By contrast, at the upper end of the distribution the relationship flattened and even showed a slight positive slope: predicted incidence rose from 5.03 to 5.58 per 1,000 person-years (Δ = +0.55) among adults aged ≥ 40 years and from 8.85 to 9.27 per 1,000 person-years (Δ = +0.42) among those aged ≥ 60 years when mean income increased from €45,987 to €55,987. Taken together, these non-linear patterns suggest a strong protective association of higher neighbourhood income at the lower end of the distribution, particularly for T2DM, with a much weaker association in the most affluent areas.

Across all models, the smooth term for mean income was statistically significant (F-statistics 9.85–78.1, *p* < 0.001) and exhibited edf greater than one (3.37–6.19), confirming clear non-linearity in the income-incidence relationship. The proportion of deviance explained ranged from modest for CVD (7–17%) to substantial for T2DM (33–51%), indicating markedly stronger socio-economic structuring in the spatial distribution of diabetes incidence.

## Discussion

In this population-based study of adults aged ≥ 40 years in Valencia (2015–2022), the estimated crude incidence was 5.85 per 1,000 person-years for CVD and 8.55 per 1,000 person-years for T2DM, with incidence increasing with age and consistently higher among men than women. Our T2DM diagnosed incidence is higher than the age- and sex-adjusted national estimate reported in the Rojo-Martínez et al. [9] cohort (3.7 cases per 1,000 person-years), which is consistent with the fact that the estimates from their study are based on population aged ≥ 18 years. The age-specific pattern observed, rising through middle and older adulthood with a decline in the oldest age groups, is in line with trends reported in Catalonia, where T2DM incidence declined in adults aged ≥ 70 years but increased in the 55–69 age group [10]. For its part, CVD incidence has shown an upward trend in recent decades, with higher rates in men than women [10], consistent with the results found in this study, and the substantial CVD burden highlighted in global projections [5, 6].

A central finding of this study is the presence of clear socioeconomic gradients in the incidence of both T2DM and CVD, measured at the census tract level using mean income per unit of consumption. While incidence remained higher among men across all income levels, the socioeconomic gradient was evident in both sexes and was consistently steeper for T2DM than for CVD. For T2DM, incidence in the lowest income quartile was approximately 60% higher than in the highest income quartile among men and nearly 90% higher among women. This pronounced socioeconomic gradient is consistent with evidence from Spain and with broader European and meta-analytic literature showing that lower SES is associated with higher T2DM incidence, as well as with greater prevalence and poorer glycaemic control [10, 13, 27, 28]. In Madrid, EHR-based analyses demonstrated a clear monotonic increase in diabetes burden with decreasing neighbourhood SES, with steeper gradients among women [13], while in Catalonia socioeconomic inequalities in diabetes incidence persisted across age and sex groups despite overall temporal declines [10]. National survey data further indicate substantial regional heterogeneity in diabetes-related socioeconomic inequalities across Spain, identifying the Comunitat Valenciana as one of the regions with the widest inequalities [12]. In contrast, although CVD incidence also displayed an inverse income gradient, differences between income groups were more modest. This attenuation is compatible with the multifactorial aetiology of CVD, in which upstream socioeconomic influences are driven through multiple intermediate risk factors – including T2DM, hypertension, dyslipidaemia, smoking, psychosocial stress, and differential access to and quality of care – each contributing to cardiovascular risk to varying degrees across social and spatial contexts [2, 29]. Previous studies have shown that socioeconomic inequalities in individual cardiovascular risk factors are often stronger than inequalities observed for CVD incidence itself, reflecting this cumulative and mediated causal structure [12, 13]. At the population level, this dilution of socioeconomic gradients at more distal disease endpoints has also been described in broader epidemiological analyses of CVD in Europe [1, 2], supporting the interpretation that socio-spatial patterning becomes less pronounced as disease pathways lengthen and diversify.

The non-linear associations further indicate that these inequalities are not distributed evenly across the full income range. In both outcomes, but especially in T2DM, the steepest differences were concentrated in the lower part of the income distribution, whereas the gradient flattened markedly in more affluent census tracts. This indicates that the marginal change in diagnosed incidence with respect to area-level income is strongest among the most socioeconomically disadvantaged census tracts. In practical terms, relatively small differences in income at the lower end of the distribution are associated with comparatively larger differences in incidence, whereas additional increases in income among more affluent areas are linked to much smaller changes. From a public health perspective, this concentration of the gradient in the lower tail of the income distribution suggests that the largest potential gains may be achieved in the most disadvantaged urban areas, where incidence appears most sensitive to socioeconomic differences.

This pattern is consistent with the possibility that the spatial distribution of diagnosed cardiometabolic incidence is shaped by the spatial distribution of socioeconomic, behavioural, and demographic drivers, as previously discussed. In urban settings, low-income areas may accumulate less favourable food environments, fewer opportunities for physical activity, and fewer health-promoting neighbourhood resources, while also concentrating populations with fewer socioeconomic resources and a higher baseline prevalence of behavioural and metabolic risk factors. Accordingly, the present findings should not be interpreted as evidence of a purely contextual neighbourhood effect, but rather as reflecting an interplay between contextual and compositional processes.

Regarding the temporal evolution of age-adjusted incidence, results showed that for both CVD and T2DM, incidence declined steadily between 2015 and 2020, followed by a partial rebound in 2021–2022. Throughout the study period, men consistently experienced higher CVD incidence than women, with a stable male-to-female ratio of around 1.8-2.0. T2DM showed a similar temporal pattern but at higher absolute levels and with a more pronounced rebound after 2020, with men having consistently higher T2DM incidence than women, although sex differences were smaller than for CVD, with male rates typically 1.3–1.6 times those of women. Overall, these trends indicate a sustained decline in diagnosed CVD and T2DM incidence up to 2020, followed by a partial reversal in the post-pandemic period. This partial reversal in age-adjusted CVD and T2DM incidence observed after 2020 is most plausibly explained by the indirect effects of the COVID-19 pandemic on healthcare use and diagnostic activity, rather than by abrupt changes in underlying disease risk. During the first year of the pandemic, widespread reductions in non-COVID healthcare use were reported, driven by lockdown measures, reallocation of resources, and patient reluctance to seek care, particularly in primary care settings where chronic disease detection typically occurs [30]. Consistent with this, several international studies documented substantial declines in incident T2DM diagnoses in 2020, including reductions of 16–33% in Finland, Canada, and Spain, attributed to restricted access to care and reduced opportunistic screening [31]. A large German study similarly observed a decrease in T2DM incidence during the initial pandemic period, followed by a marked increase in 2021, supporting the presence of a diagnostic catch up effect as healthcare use gradually normalised [32]. Comparable patterns have also been reported in Catalonia [33]. For CVD, the post-2020 increase likely reflects a combination of delayed presentations, rebound in hospital admissions, and excess mortality during the pandemic. In Spain, hospitalisations for acute coronary syndromes fell sharply during the first wave, with a 27.6% reduction in suspected ST-elevation myocardial infarctions, accompanied by longer delays to care and higher mortality [34]. International evidence indicates that confinement measures were associated with reduced CVD admissions, increased out-of-hospital cardiovascular deaths, and a subsequent rebound in hospitalisations once restrictions were lifted [35]. Together, these findings support the interpretation that the observed decline in incidence in 2020 reflects temporary underdiagnosis and altered care-seeking, while the increase in 2021–2022 represents a partial recovery of diagnostic activity in the post-pandemic period.

Beyond socioeconomic gradients, this study provides robust evidence of significant spatial clustering of incidence, particularly for T2DM. All four incidence surfaces exhibited positive spatial autocorrelation, but the magnitude differed markedly by condition. Moran’s *I* values for T2DM were more than twice those observed for CVD, indicating much stronger area-based clustering. The markedly stronger spatial autocorrelation observed for T2DM compared with CVD likely reflects the fact that T2DM lies closer to neighbourhood socioeconomic conditions and place-based exposures in the causal chain. Socioeconomic disadvantage shapes lifestyle-related risk factors, such as diet quality [36–38], obesity [39–41], and physical inactivity [42, 43], that are themselves spatially clustered within urban environments. For example, Wu et al. [18] show that spatial autocorrelation in the geographical distribution of T2DM incidence (*I* = 0.52, *p* < 0.001) is less intense than in obesity prevalence (*I* = 0.67, *p* < 0.001), keeping the territorial units of analysis constant; similarly, Zhang et al. [19] show a stronger autocorrelation in the spatial distribution of the neighbourhood deprivation index (*I* = 0.772, *p* < 0.001) than in the incidence of T2DM (*I* = 0.531, *p* < 0.001). Other studies have shown that diabetes prevalence and incidence cluster geographically in relation to built environment characteristics, including walkability, access to healthy foods, and other contextual features that structure daily behaviours [44, 45]. As a result, these socioeconomically patterned inequalities may be reflected more directly in the spatial distribution of T2DM incidence. CVD, in contrast, represents a more distal endpoint, influenced by a longer and more heterogeneous causal pathway in which upstream socioeconomic effects are mediated through multiple intermediate risk factors, including T2DM itself, and modified by prevention, treatment, survival, and competing mortality [12]. Consequently, the spatial signal originating from socioeconomic patterning becomes progressively diluted by the time it manifests as CVD incidence. This interpretation is consistent with the weaker income-incidence association and lower Moran’s *I* values observed for CVD compared to T2DM.

The fine-grained spatial scale used in this study is important for the interpretation and practical relevance of these findings. As discussed in the Introduction, analyses based on larger territorial units may obscure substantial intra-urban heterogeneity because of the MAUP, diluting local socioeconomic contrasts and masking high-burden pockets within the city. By working at census tract level, the present study provides a more detailed view of socio-spatial inequality in Valencia and helps identify areas where elevated incidence and socioeconomic disadvantage co-occur. This is precisely why the findings are relevant to the design of geographically targeted interventions: they do not merely show that inequalities exist, but indicate where prevention, screening, and community health efforts may need to be prioritised.

### Strengths and limitations

First, it should be acknowledged that this study is not based on a closed prospective cohort, but rather on a retrospectively constructed open cohort derived from RCCD. As such, the spatial-temporal observation window is explicitly delimited (see Supplementary Materials). Consequently, events occurring “under the radar” – most notably, migration into the observational frame – may lead to the misclassification of disease onsets, whereby diagnoses recorded during follow-up do not correspond to true first-ever events but to pre-existing conditions previously detected in other healthcare areas, either nationally or internationally (false positives). To mitigate this limitation, we restricted the population to adults aged ≥ 40 years, thereby reducing the inclusion of life-course ages with the highest residential mobility in a cohort defined by annual residence within the study area. In addition, we implemented a set of algorithmic rules (described in detail in the Supplementary Materials) based on the clinical plausibility of ICD-9-CM and ICD-10-CM diagnostic codes as incident events, combined with retrospective screening of individual morbidity histories, in order to minimise false positive classification. Moreover, the available spatial-temporal observation window extends beyond the formal study framework, which further contributes to reducing onset misassignment, although it cannot be fully eliminated in a study of this nature.

Second, the incidence estimates presented here reflect diagnosed incidence rather than true biological onset. Under this operational definition, false negatives may arise in two related but distinct ways. At the level of the study spatial frame, individuals may contribute a full person-year to the denominator because they appear in the annual population registry, while having effectively left the observational geographic area during part of that year or permanently exiting the frame thereafter. If incident diagnoses occur outside the observed healthcare areas or after effective exit, such events will not be captured, leading to a combination of denominator inflation and under-ascertainment of incident cases. Given the typically low rates of out-migration in older adult populations, the impact of this mechanism is likely to be negligible. More fundamentally, when incidence is defined through clinical diagnosis, systematic false negatives inevitably occur at the person-year level due to the lag between biological disease onset and clinical recognition. Individuals may harbour undiagnosed cardiometabolic disease for extended periods before receiving a formal diagnosis, meaning that true incident disease may precede recorded onset by several years. This source of misclassification reflects a conceptual limitation of using diagnosis as a proxy for incidence and lies outside the scope of the present study.

For these reasons, our estimates are not directly comparable to those obtained from closed prospective cohorts specifically designed to capture population-level incidence. Nevertheless, this study provides complementary evidence that is particularly well suited to characterising spatial and temporal patterns of disease recording and diagnosis in real world healthcare settings. Moreover, the primary objective of this research – i.e., the analysis of the spatial structure of cardiometabolic incidence and its association with SES – retains external validity, as the observed gradients and spatial patterns are consistent with findings reported in the existing literature. Furthermore, given that the rates were adjusted by age and sex, it is unlikely that the ecological associations observed can be explained by differences in the demographic composition of the population.

Finally, to the best of our knowledge, this study represents one of the most fine-grained spatial analyses conducted to date in this topic. The availability of a high resolution census tract in Spain, together with recently published income data at small area level, provides an exceptional analytical framework for examining intra-urban socioeconomic inequalities. Many previous studies have necessarily relied on larger geographical units with larger population aggregates, as mentioned above; in contrast, the present approach allows for a detailed spatial resolution of socio-spatial inequalities in cardiometabolic health.

## Conclusions

Diagnosed T2DM and CVD incidence in Valencia showed clear socioeconomic and spatial inequalities at the census tract level. These inequalities were more pronounced for T2DM, which exhibited steeper income gradients, stronger spatial clustering, and a more marked concentration of excess incidence in the least affluent areas. By contrast, CVD also followed an inverse socioeconomic gradient, but with weaker spatial structuring and more attenuated differences across income strata. The non-linear analyses further suggest that these inequalities are not distributed uniformly across the area-level income spectrum: the steepest changes in incidence were concentrated in the lower part of the income distribution, especially for T2DM, whereas the relationship flattened substantially in more affluent areas. Taken together, these findings indicate that cardiometabolic risk is not evenly distributed within the city, but organised along a clear socio-spatial pattern.

These results should not be interpreted as evidence of a purely causal area-level effect. Rather, the observed patterns are more plausibly understood as reflecting an interplay between contextual and compositional processes, whereby more disadvantaged census tracts may combine less health-promoting environments with a higher concentration of residents exposed to socioeconomic and behavioural risk factors. From a public health perspective, the fine-grained spatial scale used in this study is particularly relevant, because it allows the identification of specific urban areas where elevated incidence and socioeconomic disadvantage co-occur. In this sense, the study does not merely show that inequalities exist, but helps indicate where prevention, screening, and community health efforts may need to be prioritised. Fine-grained spatial analysis of RCCD can therefore support more equitable and geographically targeted strategies to address cardiometabolic inequalities in urban settings.

## Supplementary Information

Supplementary Material 1.

## Data Availability

The individual-level pseudonymised data used in this study are not publicly available due to data protection and dissemination restrictions agreed with the data providers. Aggregated data at census tract level and the R code scripts required to partially replicate the analyses are available as Supplementary Materials.

## Abbreviations

CVD: Cardiovascular disease
EHRs: Electronic health records
GAM: Generalised additive model
NCD: Non-communicable disease
RCCD: Routinely collected clinical data
SES: Socioeconomic status
T2DM: Type 2 diabetes mellitus
