# Supplementary material for: The spatial distribution of diagnosed Type 2 diabetes mellitus and cardiovascular disease incidence in Valencia, 2015–2022: a retrospective, registry-based study
Source: BMC Public Health. 2026 Mar 29;26:1495. doi: 10.1186/s12889-026-27160-3 (PMC13151391; doi:10.1186/s12889-026-27160-3)
Supplement: Supplementary file 1 — Supplementary Material 1. [file 12889_2026_27160_MOESM1_ESM.zip › Supp Materials_Rev_Submission 2026.03.17.docx]

**Supplementary material: The spatial distribution of diagnosed Type 2 Diabetes Mellitus (T2DM) and Cardiovascular disease (CVD) incidence in Valencia, 2015-2022: a retrospective, registry-based study.**

**Primary data characteristics, data consistency and ethical limitations**

**Primary data and internal inconsistencies**

The study relied on two individual-level administrative data systems maintained by the *Generalitat Valenciana*: the regional population register (*Sistema de Información Poblacional*, SIP) and the outpatient morbidity registry (SIA-GAIA). SIP provides pseudonymised unique health identifiers, demographic attributes (sex, date of birth, nationality), and residential information. A geocoded residence identifier at the census tract level is available in SIP only from 2015 onwards. This temporal constraint defines the period for which population-at-risk denominators can be spatially harmonised and aligned with morbidity data. SIA-GAIA contains diagnostic information coded under ICD-9-CM and ICD-10-CM and offers historical coverage back to the early 1990s, although completeness assessments based on annual record volumes indicate substantial and systematic under-recording until approximately 2005. Despite this early under-capture, the post-2005 window remains sufficiently long to ascertain pre-existing conditions among long-term residents across the six contributing health departments – Manises, València-Clínic-Malva-rosa, València-Arnau de Vilanova-Llíria, València-La Fe, València-Hospital General, and València-Doctor Peset – whose combined catchment area (Figure S1) exceeds largely the study area boundaries, thereby reducing the likelihood of misclassifying prevalent as incident cases.

Deterministic linkage across annual SIP extracts was highly stable. However, low-level inconsistencies were detected in some identifiers, most commonly one-year discrepancies in recorded year of birth and occasional discordant values in sex. To enforce intrapersonal coherence prior to the retrospective cohort construction, modal imputation was applied to both fields per identifier across all available years. Records with implausible ages (≥110 years) or missing sex, derived age group, or census tract identifiers were excluded from the analytical dataset. Census tract-level income indicator (specifically, the mean income per unit of consumption) was sourced from the National Statistics Institute’s *Atlas de Distribución de Renta de los Hogares*.


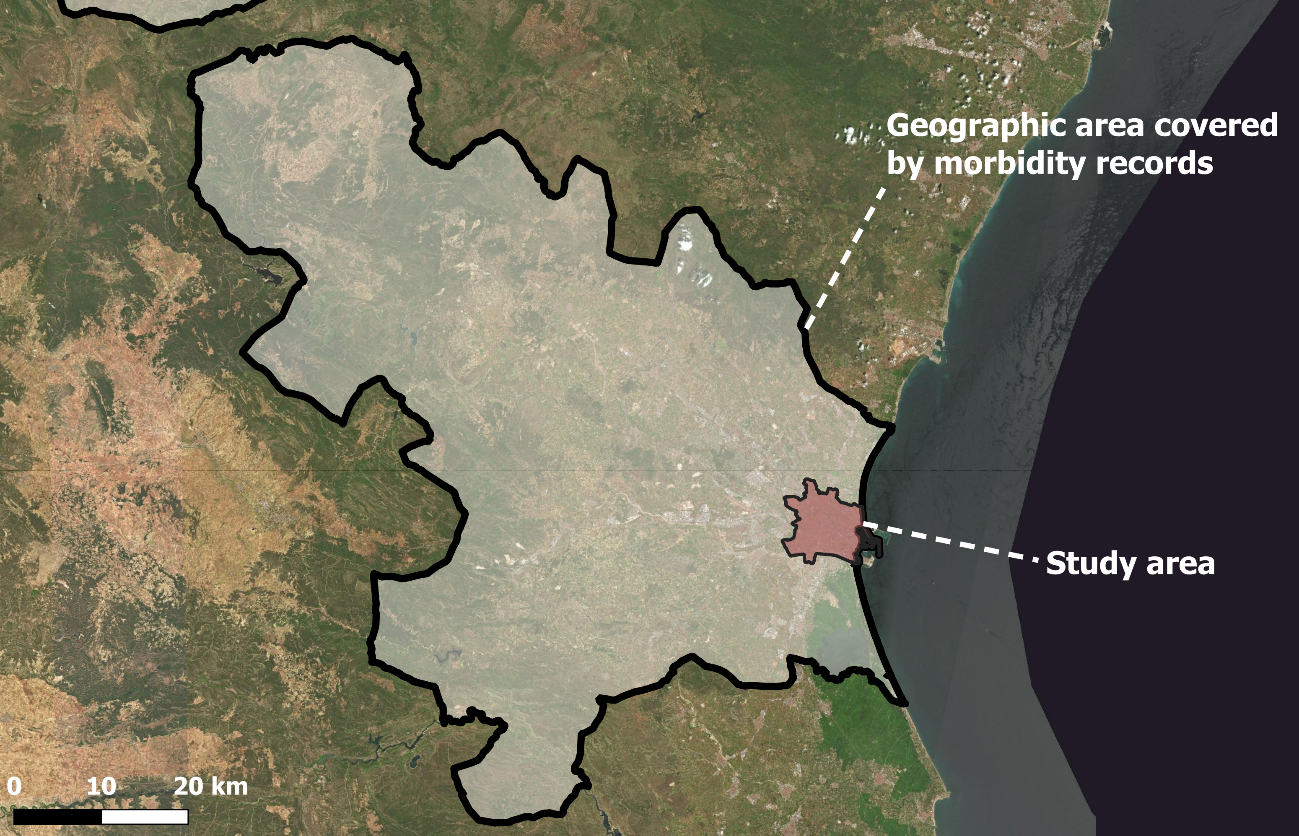


**Figure S1**. Geographical comparison between the area covered by morbidity records and the study area.

**Data management and reliability of derived variables**

All data transformations were implemented in R (version 4.3.3) through a fully scripted workflow, with intermediate artefacts persisted to disk to guarantee traceability and reproducibility. Integrity checks followed every merge, reshape or spatial operation, including verification of person-year balances, longitudinal coherence of identifiers, and one-record-per-person-year constraints. Harmonisation of population and morbidity datasets ensured a consistent longitudinal structure aligned to municipal residency. Notwithstanding these safeguards, several sources of residual error inherent to administrative data must be recognised.

First, Electronic Health Records (EHRs) -derived morbidity data are contingent on local coding practices, clinical workflows and centre-specific documentation conventions, generating the possibility of differential misclassification patterns across districts or service units. Second, temporal incongruence between biological onset and first coded diagnosis may induce left-censoring, and unobserved prior disease may persist among individuals with incomplete historical exposure to the regional health system. Such cases introduce an upward bias in incident counts despite the extended look-back window. Third, although dasymetric masking reduced the impact of large peri-urban polygons, geometric harmonisation of annually shifting census geometries to the 2022 reference inevitably entails small degrees of spatial misallocation, further compounded by short-range residential mobility near tract boundaries. Fourth, adjusted rate estimation is sensitive to sparse strata: census tracts with low person-years – particularly recently subdivided units – may exhibit instability in age- and sex-adjusted incidence despite the use of Fay-Feuer gamma limits. Due to this, three census tracts with under-recorded population were removed from the incidence maps. Finally, several internal validation layers were applied to ensure algorithmic correctness: conservation of mass checks after areal interpolation, cross-verification that each individual contributed at most one onset year, and confirmation that inclusive cut-off rules (for out-of-frame prevalence and secondary-before-primary precedence) removed all post cut-off person-time. These sources of error are predominantly systematic rather than stochastic, and were therefore addressed analytically through explicit internal validation and standardisation procedures.

**Ethics and data protection considerations**

Although both the population and morbidity datasets are pseudonymised, they remain at the individual level and contain variables that could, in combination, allow indirect re-identification under certain circumstances. The use of these data was authorised for the specific purpose of this study under a data provision agreement with the Valencian Health Authority (*Generalitat Valenciana*, SIA-GAIA system). Public release of the raw individual-level data would therefore contravene the original terms of data access and the applicable European and national data-protection regulations (GDPR and Spanish Organic Law 3/2018).
For transparency and reproducibility, we provide open access to the aggregated data outputs derived from the study – namely, cumulative incidence counts, population-at-risk and age- and sex-adjusted rates for the period 2015-2022 – at the census tract level. These spatially aggregated datasets pose no residual disclosure risk and allow independent verification of the analytical workflow and replication of all area-based analyses presented in the paper.

**Incidence**

We assembled a retrospective, open cohort for the city of Valencia (2015-2022) by deterministically linking routinely collected clinical data (RCCD) from EHRs to annual population registers using the unique health identifier. Residence was defined annually from SIP locality fields, and individuals contributed person-years only for calendar years between 2015 and 2022 in which a non-missing census tract identifier within the study area was present (“under observation”). Because census tract identifiers in the population register are only available from 2015 onwards, the observation frame was restricted to 2015-2022. Observation was organised in person-years, with one record per individual and calendar year while resident within the study area. The analytical population comprised adults aged 40-109 years with valid sex and non-missing census tract identifiers. Denominators were thus resident-years at risk, subsequently aggregated by calendar year, sex, five-year age group, and census tract. Geometries were harmonised to a 2022 baseline to ensure consistent spatial units across time (see ‘Areal interpolation’ section). The regional outpatient morbidity archive has been available since the 1990s and was searched longitudinally for each identifier, so that diagnoses recorded before 2015 or outside the study geographical area (within the boundaries defined in Figure S1) could be used to flag pre-existing disease and reduce left-censoring while not contributing person-time within the 2015-2022 frame.

Incident morbidity was identified using a two-tier coding strategy for each condition (T2DM and CVD, the latter encompassing ischaemic heart disease and cerebrovascular disease codes). First, a comprehensive screening list captured all clinically relevant ICD-9-CM/ICD-10-CM codes. Second, a prespecified subset of codes consistent with initial or acute presentations was used to operationalise incident events. This design prioritised specificity for first-ever disease while retaining transparency and reproducibility. For clarity, the complete subject-level application of the onset-assignment algorithm – including the handling of screening codes, incident-compatible codes, secondary/non-incident diagnoses, inclusive cut-offs and within-year precedence – is presented step-by-step in Annex I (Figure S.A.1), together with detailed explanatory footnotes.

To minimise misclassification from historical or delayed documentation, we applied two safeguards before assigning incidence. First, individuals with a diagnosis recorded in a calendar year when they were not under observation in the population register were treated as having pre-existing disease captured outside the cohort frame. For these persons, risk time was removed from the first subsequent year in which they (re-)appeared in the register, inclusive of that year, so that only person-years strictly prior to this inclusive cut-off contribute to denominators. Second, within-person chronology was evaluated by comparing the earliest date of any code indicative of non-incident or complication-type disease against the earliest date of a code compatible with an initial/acute presentation. If the former occurred strictly earlier by date, the individual was removed from the risk set from the calendar year of that earlier diagnosis, inclusively. When both codes fell in the same calendar year, ties were resolved by date. Only if the non-incident code preceded the incident-compatible code was the exclusion triggered, otherwise the first acute/initial code in that year was retained as an incident onset. When a primary and a secondary code shared the exact same event date, the episode was treated as incident-compatible and did not trigger secondary-based exclusion, in accordance with the precedence rules illustrated in Figure S.A.1.

Among those remaining at risk, incident cases were defined as the first occurrence of a code compatible with an initial or acute presentation during 2015-2022 while under observation. Each individual contributed at most one onset year. Person-time was accrued up to and including the onset year and was not accrued thereafter, so that no post-onset person-years enter the denominators. Annual numerators (incident onsets) and denominators (person-years at risk) were then summarised across age-sex strata by census tract for subsequent rate estimation and spatial analysis. The operational sequence underlying these rules – including onset eligibility, exclusion cut-offs, censoring, and the construction of person-time – is depicted in Annex I (Figure S.A.1).

**Case definition and coding rules for incident outcomes**

Incident events were ascertained using ICD-9-CM and ICD-10-CM diagnosis codes recorded in EHRs. Each diagnosis code was mapped to one of three phenotypic categories:

1. **Incident-compatible**. Codes judged to be clinically compatible with the first documented manifestation of the target disease in the health system. This category comprises: (i) codes that explicitly denote a new or acute presentation (e.g. a first recorded diagnosis of T2DM without chronic complications in primary care, acute myocardial infarction, acute stroke, transient ischaemic attack, or acute metabolic decompensation of diabetes); (ii) codes for chronic ischaemic heart or cerebrovascular disease in native vessels that, when recorded for the first time, typically represent the first specialist confirmation of underlying atherosclerotic disease; and (iii) a small number of “unspecified” codes that are routinely used in clinical practice at initial diagnosis before further refinement. The earliest occurrence of any incident-compatible code was taken as the index event.
2. **Secondary/complication**. Codes indicating ongoing care for an already established condition, including chronic complications, late effects, follow-up visits or subsequent episodes of care. This category includes long-term microvascular and macrovascular complications of diabetes (renal, ophthalmic, neurological, peripheral vascular, dermatological, oral and foot complications, as well as hypoglycaemic episodes), complications and sequelae of ischaemic heart disease (old or subsequent myocardial infarction, mechanical complications, graft or transplant coronary disease, post-infarction angina) and sequelae of cerebrovascular disease captured in dedicated “late effects” codes (e.g. hemiplegia, aphasia, cognitive or visual deficits following stroke). These codes were interpreted as evidence of prevalent disease when they occurred before any incident-compatible code for the same individual (or outside the observation frame) and were not used to define incident events, although they were used to describe disease burden and sequelae.
3. **Excluded**. Codes representing conditions outside the target spectrum of cardiometabolic vascular disease, that is, conditions not primarily driven by atherosclerotic, thromboembolic or hypertensive mechanisms. This category comprises congenital or structural malformations and non-atherosclerotic vasculopathies (such as unruptured intracranial or coronary aneurysms, cerebral amyloid angiopathy, Moyamoya disease, primary or secondary vasculitides, non-pyogenic cerebral venous thrombosis, non-ruptured arterial dissections and reversible or drug-induced vasoconstriction syndromes, among others). Because these entities have distinct aetiology, risk factor profiles and prognosis, they were not used either as incident events or as markers of prevalent disease in the present analyses.

This classification was applied consistently across T2DM, ischaemic heart disease (IHD) and cerebrovascular disease, with minor disease-specific refinements as detailed below. The lists of ICD-9-CM and ICD-10-CM codes that follow are therefore not intended as an exhaustive a priori catalogue of all possible diagnostic codes for these conditions, but rather describe how we classified every code that was actually observed at least once in the regional morbidity registry.

**T2DM:**

| **Incident-compatible**. Codes indicating T2DM without recorded chronic complications or with acute metabolic decompensation at or near first presentation (hyperosmolar state, diabetic ketoacidosis, or marked hyperglycaemia).  **ICD-9-CM codes**: 250.0{0,2}-250.3{0,2}.  **ICD-10-CM codes**: E11.0{0,1}, E11.1{0,1}, E11.65, E11.9.  These codes were treated as probable incident diagnosis or acute debut of T2DM.  **Secondary**. Codes denoting long-term microvascular and macrovascular diabetic complications and other late manifestations (e.g. nephropathy, retinopathy, neuropathy, peripheral angiopathy, chronic foot disease, periodontal and skin/oral complications, resolved diabetic macular oedema, or hypoglycaemic episodes in patients with established T2DM).  **ICD-9-CM codes**: 250.4{0,2}-250.9{0,2}, 357.2, 362.0 (all subcodes).  **ICD-10-CM codes**: E11.2-E11.8 (all subcodes, excluding E11.65).  These codes were interpreted as evidence of prevalent T2DM and were used only to characterise long-term disease burden and sequelae, not to define incident events. |
| --- |

**Ischaemic heart disease (IHD):**

| **Incident-compatible**. Acute coronary syndromes, unstable or stable angina, and chronic ischaemic heart disease due to atherosclerosis of native coronary arteries, including chronic total occlusion, silent ischaemia and lipid-rich or calcified plaques in native vessels (including native coronary arteries of transplanted hearts). Where episode of care subcodes were available, “initial episode of care” codes were treated as incident-compatible, whereas subsequent or unspecified episodes of care were classified as secondary.  **ICD-9-CM codes**: 410 (and subcodes, excluding unspecified or subsequent episodes of care: 410.x{0,2}), 411 (and all subcodes, excluding 411.0), 413 (and all subcodes), 414.{0,00,01}, 414.2-414.9.  **ICD-10-CM codes**: I20 (all subcodes), I21 (all subcodes), I24 (all subcodes, excluding I24.1), I25.1 (all subcodes), I25.6, I25.759, I25.811, I25.82-I25.9.  **Secondary**. Post-infarction complications, late sequelae or follow-up of a prior myocardial infarction, aneurysmal or ischaemic remodelling of the myocardium or coronary tree, and atherosclerotic disease confined to coronary bypass grafts (including grafts in transplanted hearts). These codes were interpreted as evidence of established IHD and were not used to define incident events, although they contributed to describing long-term macrovascular and myocardial sequelae.  **ICD-9-CM codes**: 410.x{0,2} (subsequent or unspecified episodes of care), 411.0, 412, 414.02-414.05, 414.07, 414.1 (all subcodes).  **ICD-10-CM codes**: I22 (all subcodes), I23 (all subcodes), I24.1, I25.{2,3}, I25.41, I25.5, I25.7{00,20,98}, I25.81{0,2}.  **Excluded**. Non-atherosclerotic coronary artery dissection, considered a distinct structural vasculopathy with different aetiology and risk factor profile from typical cardiometabolic IHD. These codes were not counted as incident events nor as markers of prevalent IHD.  **ICD-9-CM codes**: 414.12.  **ICD-10-CM codes**: I25.42. |
| --- |

**Cerebrovascular disease:**

| **Incident-compatible**. Acute cerebrovascular events including non-traumatic subarachnoid and intracerebral haemorrhage, other non-traumatic intracranial haemorrhage, cerebral infarction, acute occlusion/stenosis of precerebral or cerebral arteries, and acute or ill-defined cerebrovascular disease. Transient ischaemic attacks (TIA) were also included as probable incident events when coded as first occurrence.  **ICD-9-CM codes**: 430-436 (and all subcodes), 437, 437.{0,1,9}.  **ICD-10-CM codes**: I60-I66 (all subcodes, excluding I63.6), I67.{2,3}, I67.8{1,2}, I67.9.  **Secondary**. Sequelae and other late effects of cerebrovascular disease, including residual motor deficits (hemiplegia, monoplegia, other paralytic syndromes), cognitive and language impairment, sensory loss, visual disturbance and other chronic post-stroke symptoms; these were treated as prevalent rather than incident events.  **ICD-9-CM codes**: 437.{2,8}, 438 (and all subcodes).  **ICD-10-CM codes**: I67.4, I67.89, I69 (all subcodes).  **Excluded**. Vascular abnormalities and other cerebrovascular conditions that do not themselves represent an acute stroke event (e.g. unruptured aneurysm, hereditary arteriopathy, vasculitis, non-pyogenic venous sinus thrombosis, reversible vasoconstriction/vasospasm syndromes, cerebral amyloid angiopathy, and cerebrovascular involvement coded only as part of other systemic diseases).  **ICD-9-CM codes**: 437.3-437.7.  **ICD-10-CM codes**: I63.6, I67.{0,1,5,6,7}, I67.8{3,41,48,50}, I68 (and subcodes). |
| --- |

**Case definition sensitivity and misclassification of incident events**

Using RCCD from EHRs to define incident events inevitably raises concerns about misclassification. Despite the long historical coverage of the regional morbidity archive and the use of extended look-back windows to identify prior diagnoses, some onset events will be imperfectly captured. Individuals moving into or out of the region, or receiving part of their care in other health systems, may have relevant diagnoses recorded outside the data sources available to this study. When such patients first appear in the regional EHR, their earliest recorded code may represent a late recording of long-standing disease rather than a true debut. In addition, coding practices vary across clinicians, services and over time: some professionals may preferentially code generic or “unspecified” diagnoses at first contact and refine them later, whereas others may directly register specific phenotypes or chronic sequelae. Acute conditions can be recorded either as explicit incident events (e.g. acute myocardial infarction, acute stroke, newly diagnosed type 2 diabetes without complications) or indirectly via related codes such as “old” infarction, late effects of stroke, longstanding microvascular complications of diabetes or hyperglycaemic crises. These patterns create a tension between under-ascertainment of incident disease (if only unequivocal acute-onset codes are used) and over-ascertainment (if codes that often reflect long-standing disease are treated as incident). Our primary case definition seeks a clinically plausible compromise, but some residual misclassification is unavoidable.

To assess the robustness of our findings to these uncertainties, we re-estimated incidence under two alternative case definition scenarios that modify the mapping between ICD codes and the “incident-compatible” category, keeping the underlying cohort construction, wash-out and censoring rules unchanged.

| **Type** | **Scenario** | **Codes (ICD-9-CM/ICD-10-CM)** |
| --- | --- | --- |
| CVD | Conservative | **(kept)**: 410.x1 (initial episode of care), 430-432 (and all subcodes), 433.x1, 434.x1, I21 (all subcodes), I24.0, I60-I63 (all subcodes, excluding I63.6). |
|  | Sensitive | **(added)**: 410.x0 (episode of care unspecified), 437.2, 437.8, I67.4, I67.89. |
| T2DM | Conservative | **(kept)**: 250.0{0,2}, E11.9. |
|  | Sensitive | **(added)**: 250.4{0,2}-250.9{0,2}, 357.2, 362.0 (all subcodes), E11.2-E11.8 (and all subcodes; E11.65 was already included in the main list). |

The resulting incidence estimates were broadly consistent across scenarios, and their comparison illustrates the plausible range of misclassification arising from coding choices. For CVD in the main scenario definition, we identified 19,902 first incident events over 3,401,388 person-years at risk, yielding a crude incidence rate of 5.85 per 1,000 person-years. Under the sensitive scenario, the number of incident CVD events increased modestly to 20,523 over 3,400,125 person-years at risk, corresponding to 6.04 per 1,000 person-years. This reflects a small number of additional cases captured when late-effect or borderline codes are allowed to signal onset. In the conservative scenario, which limits incident-compatible codes to the most unequivocal acute or debut diagnoses, 10,485 CVD events were detected over 3,423,887 person-years, giving a crude incidence rate of 3.06 per 1,000 person-years – approximately half the estimate obtained in the main analysis and consistent with a lower bound that sacrifices sensitivity to minimise the inclusion of long-standing disease. For T2DM, the main scenario definition yielded 27,245 incident events during 3,187,938 person-years at risk (8.55 per 1,000 person-years). The sensitive scenario, which allows for debuts documented via complications or crises, produced 32,746 incident T2DM cases over 3,175,993 person-years, increasing the crude rate to 10.30 per 1,000 person-years. The conservative scenario, in which only the most specific debut codes are considered, identified 25,874 cases over 3,191,848 person-years (8.11 per 1,000 person-years), a modest reduction relative to the main estimate. Taken together, these analyses suggest that our main scenario definition yields CVD and T2DM incidence rates that lie between a stringent lower bound and a more inclusive, practice-sensitive upper bound, and that the main substantive patterns reported in this paper are robust to clinically plausible alternative assumptions about how EHR codes signal incident disease.

**Areal interpolation**

The study area encompasses 26 postal codes within the urban fabric of Valencia, corresponding to a spatial footprint and resident population slightly smaller than the administrative municipality (Figure S2), yet covering the great majority of the consolidated urban core. For cartographic visualisation, spatial autocorrelation tests and area-level analyses between socioeconomic status and incidence, data were aggregated at census tract level. In the Spanish territorial hierarchy, the census tract represents the smallest spatial unit for the dissemination of official statistics – such as census and socioeconomic indicators – and also serves as the basic electoral unit. These tracts are delineated by morphologically recognisable physical boundaries (e.g., streets, rivers, railway lines) and typically encompass local populations ranging from approximately 500 to 2,500 inhabitants, thus providing a fine-grained spatial resolution for urban health analysis. Census tract boundaries are not fixed but may be periodically revised to reflect demographic change and shifts in the socio-territorial structure of the area.


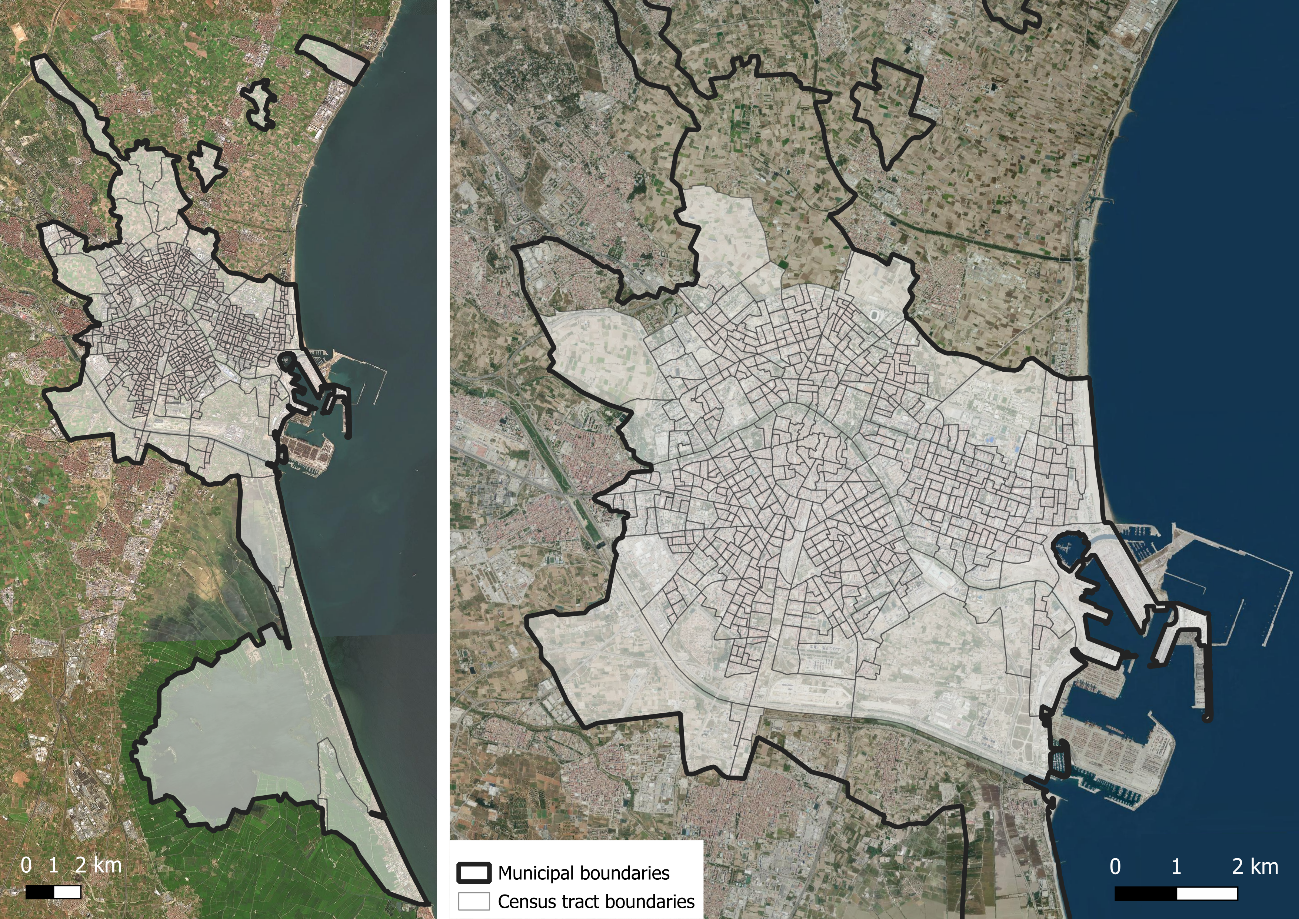


**Figure S2**. **Left**: Overview of the territorial boundaries of the municipality of Valencia. **Right**: Detailed view of the study area (three census tracts were removed from the visualization due to population under-recording). Census tracts corresponding to 2022.

The spatio-temporal comparison of epidemiological counts is compromised by shifting administrative boundaries (i.e., census tract redesigns), which introduce a bias of temporal boundary misalignment (e.g., see Figure S3). Geometric harmonisation avoids attributing observed changes to mere boundary modifications, allowing inter-annual comparisons to reflect actual epidemiological variation rather than cartographic artefacts. To obtain consistent series at the census tract level and avoid artefacts due to shifting boundaries, we harmonised all attributes of interest (i.e., annual counts of incident cases and denominators in person-years per stratum) to a single target geometry (2022) using area-weighted interpolation with dasymetric masking (Figure S4). Masking reduces the potential mis-weighting of changing peri-urban census tracts, which are typically larger in size, and improves the internal validity of the ecological assumption of uniform intra-polygon density within the study area.


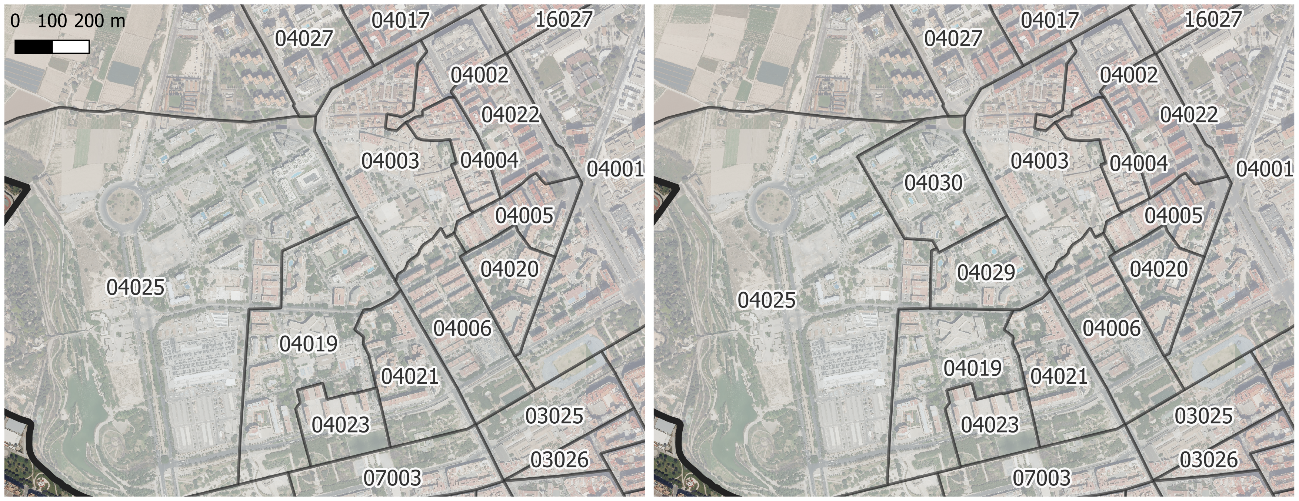


**Figure S3**. Comparison of the shifting census boundaries between 2021 (**left**) and 2022 (**right**).


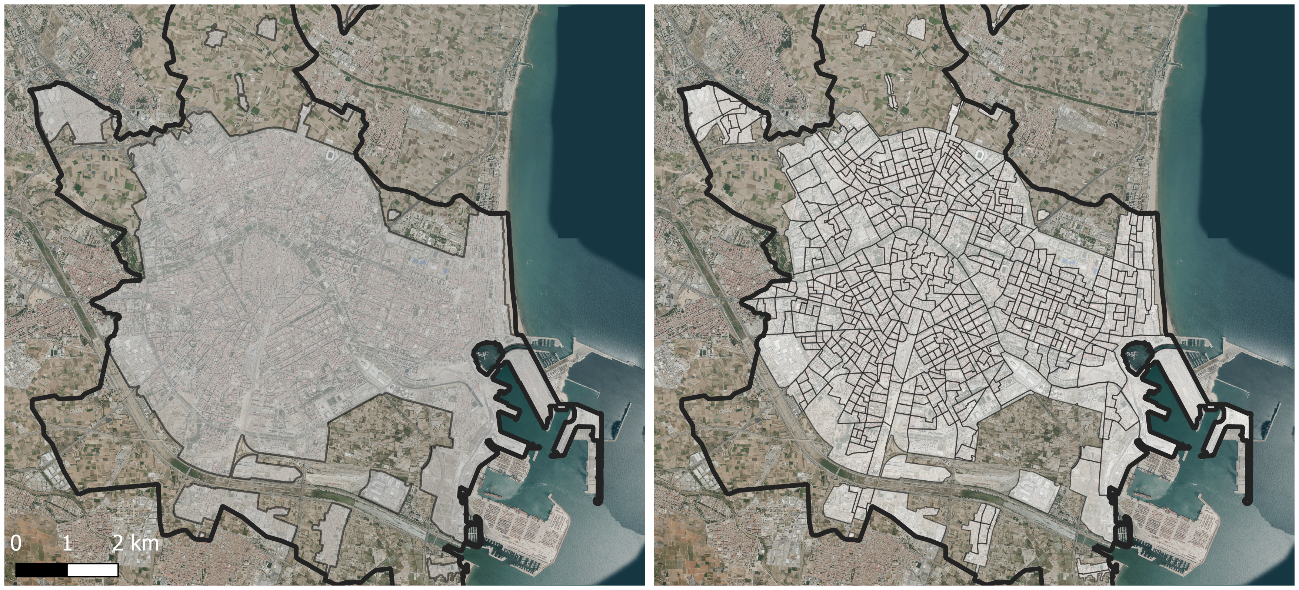


**Figure S4**. **Left**: Shape of urban land within the municipality of Valencia. **Right**: Dasymetric shape of the census tract (2022), generated by intersection with the shape of urban land.

Before interpolation, all annual layers were cropped with a dissolved vector mask delimiting the built-up area in the target year (2022; see Figure S4), in order to exclude non-built areas in large peri-urban polygons which, due to their size, distort the overlap fractions. This dasymetric approach restricts the weighting to the effectively urbanised area, reducing intra-polygonal heterogeneity that is irrelevant to the population-at-risk. Then, we spatially projected the counts and population-at-risk for each annual source geometry (from 2015 to 2021) onto a single target geometry (2022) using geometric weighting by area. Let $S$ be the set of source polygons and $T$ the set of target polygons. For each overlapping pair $(s,t)$ we computed the weight $\omega_{s\to t}=\left| s\cap t \right|\left| s \right|^{-1}$ and then renormalised by source so that $\sum_{t} \omega_{s\to t}=1$. Each attribute $x(s)$ was transferred as a weighted sum

$$y\left( t \right)=\sum_{s\in S} \omega_{s\to t}x\left( s \right),$$

which ensures mass conservation (city-wide totals are preserved). For rate calculation, this is algebraically analogous to the geometric method described by Pavía & López-Quílez (2013). As a limitation, this approach does not correct for sub-tract heterogeneity not aligned with boundaries. The R code used to implement the function is provided below:

# geometric_interpolation.R
# ----------------------------------------
# Function to perform geometric interpolation of multiple attributes
# from a source polygon layer to a target polygon layer,
# weighted by the proportion of overlapping area.
#
# Particularly useful for interpolating extensive attributes (e.g., counts)
# when territorial units are redrawn over time. Assumes coterminous outer boundaries
# (i.e., union(source) = union(target) within the study domain) so only internal
# tract boundaries change. Consequently, every source unit has non-zero overlap with
# the target geometry (no orphan sources) and area-weighted interpolation is
# mass-preserving.
#
# Ensures that for each source unit the sum of weights = 1.
#
# Requirements:
# - sf
# - dplyr

#' Geometric interpolation of numeric attributes from one sf to another
#'
#' @param source_sf sf object with original polygons and attributes
#' @param target_sf sf object defining the new polygons
#' @param interp_cols character vector of column names in source_sf
#' @param output_names character vector of names for the interpolated columns
#' @param source_id name of the unique ID field in source_sf
#' @param target_id name of the unique ID field in target_sf

geometric_interpolation <- function(
 source_sf, target_sf,
 interp_cols, output_names,
 source_id, target_id
 ) {
 # Rename ID fields
 source_sf <- source_sf %>% rename(src = all_of(source_id))
 target_sf <- target_sf %>% rename(tgt = all_of(target_id))

 # Intersection and filter polygons
 inter <- st_intersection(source_sf, target_sf) %>%
 filter(st_geometry_type(.) %in% c("POLYGON","MULTIPOLYGON"))

 # Compute area fractions
 inter <- inter %>%
 mutate(
 src_area = st_area(source_sf)[match(src, source_sf$src)],
 int_area = st_area(geometry),
 frac = as.numeric(int_area / src_area)
 ) %>%
 group_by(src) %>%
 mutate(frac = frac / sum(frac, na.rm = TRUE)) %>%
 ungroup()

 # Compute weighted sums and aggregate
 result <- inter %>%
 mutate(across(all_of(interp_cols), as.numeric)) %>%
 mutate(across(all_of(interp_cols), ~ .x * frac, .names = "w_{.col}")) %>%
 group_by(tgt) %>%
 summarise(
 across(starts_with("w_"), sum, na.rm = TRUE),
 geometry = st_union(geometry),
 .groups = "drop"
 ) %>%

 rename_with(~ output_names, starts_with("w_")) %>%

 mutate(!!target_id := tgt) %>%
 select(all_of(target_id), all_of(output_names), geometry)

 result
}

# Example:
# vlc_secc2015 <- st_read("data/vlc_secc2015.shp")
# vlc_secc2022 <- st_read("data/vlc_secc2022.shp")
# out <- geometric_interpolation(
# vlc_secc2015, vlc_secc2022,
# interp_cols = c("POPTOTAL2015"),
# output_names = c("pop2015"),
# source_id = "CUSEC", target_id = "CUSEC"
# )
# st_write(out, "output/interpolated.shp")
#

**Illustrative examples**:

1. In the raw data, census tract 04025 accumulates 61 first CVD diagnoses over 2015-2022 and the new section 04030 (segregated from 04025 in 2022) has 0 cases in 2022. In the harmonised 2022 geometry, area-weighted interpolation reallocates these 61 cases between the parent 04025 (49 cases) and the new child 04030 (12 cases), in proportion to the child’s ≈ 22% share of the original tract’s area.
2. Census tract 04019 accumulated 43 CVD incident cases and 9,861 person-years between 2015 and 2022. In 2022 it was subdivided and a new census tract, 04029, was created, occupying 33.4% of the original area. The interpolation allocates 33.4% of the pre-2022 history of 04019 to 04029 (≈13 cases and 3,072 person-years) and then adds the child tract’s own 2022 contribution (2 cases and 795 person-years), yielding a total of 15 interpolated cases and 3,867 person-years for 04029 – exactly the values used in the maps.

See Figure S3 and census tract-level estimates in the supplementary data.

**Statistical analysis**

Crude incidence rates were calculated as the ratio between the number of new onsets and the corresponding population-at-risk (×1,000 person-years). Because these rates represent directly observed frequencies derived from complete administrative population data rather than from a probabilistic sample, confidence intervals were not reported for crude rates. They describe the empirical distribution of events rather than an estimate subject to sampling variability.

Age- and sex-adjusted incidence rates were computed using the method of direct standardisation, with the city-wide population distribution as the standard. For each disease and year, the number of incident cases and the population denominators were cross-classified by sex and five-year age group, and adjusted rates (×1,000 person-years) were derived using the *epi.directadj()* function from the *epiR* package. This procedure yields Fay-Feuer gamma confidence intervals, which account for the skewness inherent in Poisson-distributed counts and provide exact coverage even in strata with low case numbers (for a more detailed description, please consult the technical notes). All annual counts and person-years were first harmonised to the 2022 census tract geometry using area-weighted geometric interpolation to ensure temporal comparability across changing administrative boundaries. Age- and sex-adjusted rates and their 95% confidence intervals were then computed on this harmonised geometry. For annual series, the age distribution of the 2022 total population-at-risk was used as the standard.

Spatial patterns of known disease incidence were characterised using these age- and sex-adjusted rates derived through direct standardisation and mapped as choropleths, with class intervals defined by natural breaks (Jenks). Global spatial autocorrelation was assessed using Moran’s I computed on queen contiguity weights with a permutation test (999 simulations) to obtain empirical p-values. For a more detailed description, please consult the technical notes.

Bivariate associations between adjusted census tract-level incidence and the socioeconomic indicator (the mean income per unit of consumption in 2022) were explored through scatterplots fitted with Generalised Additive Models (GAMs) as implemented in the *mgcv::gam()* function. Each model used as its outcome one of four census tract-level incidence measures, defined by condition (CVD or T2DM) and age threshold (≥40 or ≥60 years). The sole explanatory variable was a univariate smooth term representing the mean income per unit of consumption in 2022. This modelling strategy permits flexible, data-driven departures from linearity in the income-incidence association while preserving a transparent and readily interpretable functional form.

The smooth terms were estimated using penalised regression splines with automatic smoothness selection, where the smoothing parameter is chosen by generalised cross-validation criterion (GCV). This approach balances fidelity to the data with the imposition of smoothness, preventing overfitting while allowing the function to capture genuine curvature. The effective degrees of freedom (edf) associated with each smooth term indicate the realised flexibility of the fitted curve: values noticeably greater than 1 confirm a departure from linearity, with higher values reflecting more pronounced curvature.

For each model, we extracted the smooth term’s edf, reference degrees of freedom, F-statistic and *p*-value from the GAM summary, alongside global model diagnostics such as deviance explained and the pseudo-R². These quantities provide a concise characterisation of how strongly and how non-linearly neighbourhood income relates to incidence in each age group and disease category. Predicted incidence values were then computed for specific points along the observed income distribution (minimum to minimum + €10,000; maximum − €10,000 to maximum), allowing us to quantify the change in predicted incidence associated with income contrasts of epidemiological interest. Finally, for visualisation, we generated scatterplots with fitted GAM smoothers and 95% confidence bands to summarise the estimated functional forms across all four outcomes.

All analyses were performed in R (version 4.3.3), within a fully scripted and reproducible workflow. For Moran’s *I*:

***## Install required packages if missing***

*# pkgs <- c("readxl", "sf", "dplyr", "spdep")*
*# install.packages(setdiff(pkgs, rownames(installed.packages())))*

*# Run only if the required packages are not installed*


***## Load required packages***
**library**(readxl)
**library**(sf)
**library**(dplyr)
**library**(spdep)

***## 1. Read data --------------------------------------------------------***
*# 1.1. Read the Excel file*
*# Specify the path where you downloaded the Excel file*
rate_data <- **read_excel**("C:/write/your/path/rate_data.xlsx")

*# 1.2. Read the shapefile*
*# Same as in the previous step*
vlc_sf <- **st_read**("C:/ write/your/path/VLC_2022.shp")

***## 2. Merge census tract geometries with incidence data ----------------***
vlc_data <- vlc_sf **%>%**
 **inner_join**(rate_data, by = "CUSEC")

***## 3. Construct queen contiguity spatial weights -----------------------***
nb_queen <- **poly2nb**(vlc_data, queen = TRUE)
lw_queen <- **nb2listw**(nb_queen, style = "W", zero.policy = TRUE)

***## 4. Moran’s I with permutation test for each variable ----------------***
**set.seed**(1234)

vars_moran <- **c**(
 "CVD_40_rate",
 "CVD_60_rate",
 "DBT_40_rate",
 "DBT_60_rate"
 )

*# Store full moran.mc objects*
moran_list <- **vector**("list", **length**(vars_moran))
**names**(moran_list) <- vars_moran

**for** (v **in** vars_moran) {
 x_var <- vlc_data[[v]]

 moran_list[[v]] <- **moran.mc**(
 x = x_var,
 listw = lw_queen,
 nsim = 999,
 alternative = "two.sided"
 )
}

***## 5. Display full Moran’s I outputs -----------------------------------***
**for** (v **in** vars_moran) {
 **cat**("**\n**=====================================**\n**")
 **cat**("Moran's I Monte Carlo test for:", v, "**\n**")
 **cat**("=====================================**\n\n**")
 **print**(moran_list[[v]])
}

For fitting GAMs and scatter plots:

***## Install required packages if missing***

*# pkgs <- c("readxl", "dplyr", "tibble", "ggplot2",*
*# "mgcv", "cowplot", "scales", "patchwork")*
*# install.packages(setdiff(pkgs, rownames(installed.packages())))*

*# Run only if the required packages are not installed*


***## Load required packages***
**library**(readxl)
**library**(dplyr)
**library**(tibble)
**library**(ggplot2)
**library**(mgcv)
**library**(cowplot)
**library**(scales)
**library**(patchwork)

*# Assuming that sf has yet been loaded and vlc_data exists from the previous chunk*

***## 1. Read income data and merge with tract-level incidence -------------***

*# 1.1. Read the Excel file containing mean income per unit of consumption (2022)*
income_2022 <- **read_excel**("C:/write/your/path/MIUC_2022.xlsx")

*# 1.2. Merge income with the existing census tract dataset*
vlc_data <- vlc_data **%>%**
 **left_join**(income_2022, by = "CUSEC")

***## 2. Fit univariable GAMs for each incidence measure -------------------***

incidence_vars <- **c**("CVD_40_rate", "CVD_60_rate",
 "DBT_40_rate", "DBT_60_rate")

contrast_list <- **vector**("list", **length**(incidence_vars))
model_list <- **vector**("list", **length**(incidence_vars))

plot_data <- **list**()
gam_models <- **list**()

**for** (i **in** **seq_along**(incidence_vars)) {
 var <- incidence_vars[i]

 *# Keep tracts with non-missing incidence and income*
 df <- vlc_data **%>%**
 **filter**(**!is.na**(.data[[var]]),
 **!is.na**(.data[["MIUC_2022"]]))

 *# GAM with smooth term for the mean income per unit of consumption (MIUC_2022)*
 form <- **as.formula**(**paste**(var, "~ s(MIUC_2022)"))
 mod <- **gam**(form, data = df)

 plot_data[[var]] <- df
 gam_models[[var]] <- mod

 ***## (A) Rate contrasts for €10,000 income intervals --------------------***
 rng <- **range**(df**$**MIUC_2022, na.rm = TRUE)
 x_min <- rng[1]
 x_min2 <- **min**(rng[1] **+** 10000, rng[2])
 x_max <- rng[2]
 x_max2 <- **max**(rng[2] **-** 10000, rng[1])

 y_min <- **as.numeric**(**predict**(mod, newdata = **data.frame**(MIUC_2022 = x_min)))
 y_min2 <- **as.numeric**(**predict**(mod, newdata = **data.frame**(MIUC_2022 = x_min2)))
 y_max <- **as.numeric**(**predict**(mod, newdata = **data.frame**(MIUC_2022 = x_max)))
 y_max2 <- **as.numeric**(**predict**(mod, newdata = **data.frame**(MIUC_2022 = x_max2)))

 delta_low <- y_min2 **-** y_min
 delta_high <- y_max **-** y_max2

 contrast_list[[i]] <- **tibble**(
 variable = var,
 segment = **c**("min → min+10k", "max-10k → max"),
 x0 = **c**(x_min, x_max2),
 x1 = **c**(x_min2, x_max),
 rate_x0 = **c**(y_min, y_max2),
 rate_x1 = **c**(y_min2, y_max),
 delta_rate = **c**(delta_low, delta_high)
 )

 ***## (B) Summary statistics for the smooth term and model fit ----------***
 sm <- **summary**(mod)
 s_tab <- sm**$**s.table[1, , drop = TRUE]

 edf <- **as.numeric**(s_tab["edf"])
 ref_df <- **as.numeric**(s_tab["Ref.df"])
 F_stat <- **as.numeric**(s_tab["F"])
 p_value <- **as.numeric**(s_tab["p-value"])

 dev_expl <- sm**$**dev.expl
 r_sq <- sm**$**r.sq
 n_obs <- **nrow**(df)
 k_basis <- mod**$**smooth[[1]]**$**bs.dim

 model_list[[i]] <- **tibble**(
 variable = var,
 n = n_obs,
 edf = edf,
 ref_df = ref_df,
 F_stat = F_stat,
 p_value = p_value,
 dev_expl = dev_expl,
 r_sq = r_sq,
 k_basis = k_basis
 )
}

*# Combined outputs*
detailed_results <- **bind_rows**(contrast_list)
model_summary <- **bind_rows**(model_list)

detailed_results
model_summary

***## 3. Scatterplots with GAM smoothers ----------------------------------***

plot_colour <- "#4A6FE3"
plot_ribbon <- **alpha**(plot_colour, 0.4)

make_gam_scatter <- **function**(df, mod, var_name, title_text = NULL) {

 rng <- **range**(df**$**MIUC_2022, na.rm = TRUE)
 pred_df <- **data.frame**(MIUC_2022 = **seq**(rng[1], rng[2], length.out = 300))
 preds <- **predict**(mod, newdata = pred_df, se.fit = TRUE)
 pred_df**$**fit <- preds**$**fit
 pred_df**$**lower <- preds**$**fit **-** 1.96 ***** preds**$**se.fit
 pred_df**$**upper <- preds**$**fit **+** 1.96 ***** preds**$**se.fit

 **ggplot**(df, **aes**(x = MIUC_2022, y = .data[[var_name]])) **+**
 **geom_point**(colour = "grey50", alpha = 0.5, size = 1.4) **+**
 **geom_ribbon**(data = pred_df,
 **aes**(x = MIUC_2022, ymin = lower, ymax = upper),
 inherit.aes = FALSE,
 fill = plot_ribbon, alpha = 0.4, colour = NA) **+**
 **geom_line**(data = pred_df,
 **aes**(x = MIUC_2022, y = fit),
 inherit.aes = FALSE,
 colour = plot_colour, linewidth = 0.9) **+**
 **scale_x_continuous**(labels = comma) **+**
 **labs**(title = title_text) **+**
 **theme_classic**() **+**
 **theme**(
 axis.title.x = **element_blank**(),
 axis.title.y = **element_blank**(),
 plot.title = **element_text**(hjust = 0, size = 10, margin = **margin**(b = 6))
 )
}

p_CVD40 <- **make_gam_scatter**(
 plot_data[["CVD_40_rate"]],
 gam_models[["CVD_40_rate"]],
 "CVD_40_rate",
 "A. CVD, aged ≥40 years"
)

p_CVD60 <- **make_gam_scatter**(
 plot_data[["CVD_60_rate"]],
 gam_models[["CVD_60_rate"]],
 "CVD_60_rate",
 "B. CVD, aged ≥60 years"
)

p_DBT40 <- **make_gam_scatter**(
 plot_data[["DBT_40_rate"]],
 gam_models[["DBT_40_rate"]],
 "DBT_40_rate",
 "C. T2DM, aged ≥40 years"
)

p_DBT60 <- **make_gam_scatter**(
 plot_data[["DBT_60_rate"]],
 gam_models[["DBT_60_rate"]],
 "DBT_60_rate",
 "D. T2DM, aged ≥60 years"
)

grid_plot <- (p_CVD40 **|** p_CVD60) **/** (p_DBT40 **|** p_DBT60)
grid_plot <- grid_plot **&** **theme**(plot.margin = **margin**(t = 5, r = 5, b = 12, l = 10))

final_scatter_gam <- **ggdraw**(grid_plot) **+**
 **draw_label**("Age- and sex-adjusted incidence (×1,000 person-years)",
 x = 0.02, y = 0.5, angle = 90, size = 11) **+**
 **draw_label**("Mean income per unit of consumption in 2022 (€)",
 x = 0.5, y = 0.02, size = 11)

final_scatter_gam

**Technical notes**

**Gamma confidence limits for directly standardised rates**

The gamma confidence limits for directly standardised rates proposed by Fay and Feuer (1997) approximate the sampling distribution of a weighted sum of Poisson rates by a gamma distribution with the same first two moments. This method is implemented in R within the function *epi.directadj()* from the *epiR* package (Stevenson et al.), which computes the adjusted rate and its corresponding gamma-based confidence limits following the Fay-Feuer formulation. Let

$$Y=\sum_{k=1}^{K} w_{k}\frac{X_{k}}{T_{k}} ,$$

where $X_{k}$​ denotes the number of events in stratum $k$, $T_{k}$​ the corresponding person-time, and $w_{k}$ the standard population weights satisfying $\sum_{k} w_{k}=1$. The estimator $Y$ represents the directly standardised rate, i.e. the expected number of events per unit of person-time if the study population had the age-sex composition of the chosen standard population.

Fay and Feuer showed that the distribution of $Y$ can be approximated by a gamma random variable $Z\sim G(a,b)$ with mean and variance equal to those of $Y$, that is $E(Z)=E(Y)=y$ and $Var(Z)=Var(Y)=v$. Under this assumption, approximate $100(1-\alpha)\%$ confidence limits for the true mean rate $\mu$ are obtained as

$$L\left( y,v \right)=G_{\left( \frac{y^{2}}{v}, \frac{v}{y} \right)}^{-1}\left( \frac{\alpha}{2} \right) , U\left( y,v,w_{m} \right)=G_{\left( \frac{\left( y+w_{m} \right)^{2}}{v+w_{m}^{2}}, \frac{v+w_{m}^{2}}{y+w_{m}} \right)}^{-1}\left( 1-\frac{\alpha}{2} \right) ,$$

where $G_{\left( a,b \right)}^{-1}(p)$ denotes the $p$-quantile of a gamma distribution with shape $a$ and scale $b$. The term $w_{m}={max}_{k}(w_{k}/T_{k})$ represents the maximum weight–time ratio across strata and is used to derive a conservative upper confidence limit when strata have unequal weights or small counts.

In this formulation:

$y$ is the observed directly standarised rate;

$v=\sum_{k} w_{k}^{2}{X_{k}}/{T_{k}^{2}}$ is the estimated variance of $Y$;

$w_{m}$ corrects the upper bound for discreteness in the weighted sum;

$\alpha$ determines the nominal confidence level $1-\alpha$;

$G^{-1}$ ensures the limits respect the positive and asymmetric nature of the gamma distribution, providing accurate coverage even when event counts are small.

Overall, this method yields asymmetric confidence intervals that are exact in the Poisson case and maintain close to nominal coverage for standardised rates, outperforming normal approximations particularly in sparse data or highly unbalanced weighting schemes.

**Global Moran’s** $\boldsymbol{I}$ **for spatial autocorrelation**

Moran’s $I$ is the most widely used indicator of global spatial autocorrelation. It was first introduced by Moran (1948) and later popularised through the seminal work of Cliff and Ord (1973) on the theory and application of spatial autocorrelation.

Given $N$ areal units (e.g., census tracts) with a variable $x_{i}$ observed on each unit $i\neq j$ and a spatial weights matrix $W=(w_{ij})$ with zero diagonal and total weight $S_{0}=\sum_{i} \sum_{j} w_{ij}$​, the global Moran’s $I$ is

$$I=\frac{N}{S_{0}}\frac{\sum_{i=1}^{N} \sum_{j=1}^{N} w_{ij}(x_{i}-\bar{x})(x_{j}-\bar{x})}{\sum_{i=1}^{N} {(x_{i}-\bar{x})}^{2}}, \bar{x}=\frac{1}{N}\sum_{i=1}^{N} x_{i} ,$$

which represents a normalised cross-product between a variable and its spatial lag, quantifying the overall degree of spatial clustering or dispersion. Positive $I$ indicates spatial clustering of similar values (high-high / low-low), while negative $I$ indicates spatial dispersion (high-low contrasts).

Statistical inference for Moran’s $I$ can rely on analytical moments of the randomisation or normality distribution. However, an increasingly preferred and more robust approach is based on permutation inference. In this computational framework, the reference distribution of Moran’s $I$ under the null hypothesis of spatial randomness is generated by repeatedly permuting the observed values across spatial units while keeping the spatial weights fixed. Each permuted dataset yields a simulated statistic, and the resulting empirical distribution is used to estimate pseudo-$p$ values. This non-parametric procedure is less sensitive to violations of distributional assumptions, providing finite-sample valid (conservative) p-values.

In the case of a two-sided permutation test, the function computes the rank $r$ of the observed statistic $I^{obs}$ among the $M+1$ values (the $M$ permuted plus the observed one) and derives the pseudo-$p$ value from its distance to the centre of the permutation distribution. Specifically, this distance is defined as

$$D=\frac{\left| r-\left( M+1 \right)/2 \right|}{M+1} ,$$

which represents the scaled absolute deviation of the observed statistic from the median rank of the permutation distribution. Under the null hypothesis of spatial randomness, this distance $D$ follows approximately a $Uniform(0,0.5)$ distribution. The two-sided pseudo-$p$ value is thus obtained as $p_{two-sided}=Pr\left( D^{'}\geq D \right)$ where $D'$ denotes a random variable uniformly distributed on [0,0.5]. This formulation is equivalent to doubling the smaller one-sided tail while applying the standard $(R+1)/(M+1)$ correction to avoid zero $p$-values.

This permutation-based inference framework is implemented through the *moran.mc()* function of the *spdep* package (Bivand et al., 2001–2024), which computes Moran’s $I$ by repeated random permutation of the data values across spatial locations and derives pseudo-$p$ values from the resulting empirical reference distribution under spatial randomness.

**References**

Bivand R. *spdep: Spatial Dependence: Weighting Schemes, Statistics* [R package]. Version 1.4-1. 2025. Available from: https://CRAN.R-project.org/package=spdep. https://doi.org/10.32614/CRAN.package.spdep

Cliff AD, Ord JK. *Spatial autocorrelation*. London: Pion; 1973.

Fay MP, Feuer EJ. Confidence intervals for directly standardized rates: a method based on the gamma distribution. *Statistics in Medicine* 1997;16(7):791–801. https://doi.org/10.1002/(SICI)1097-0258(19970415)16:7%3C791::AID-SIM500%3E3.0.CO;2-%23

Moran PAP. The interpretation of statistical maps. *Journal of the Royal Statistical Society. Series B (Methodological)* 1948;10(2):243–51. Available from: http://www.jstor.org/stable/2983777

Pavía JM, López-Quílez A. Spatial vote redistribution in redrawn polling units. Journal of the *Royal Statistical Society Series A: Statistics in Society* 2013;176(3):655–678. https://doi.org/10.1111/j.1467-985X.2012.01055.x

Stevenson M, Sergeant E, Heuer C, Nunes T, Marshall J, Sanchez J, et al. *epiR: Tools for the Analysis of Epidemiological Data* [Internet]. Version 2.0.88. CRAN; 2025 Oct 21 [cited 2025 Oct 22]. Available from: https://CRAN.R-project.org/package=epiR. https://doi.org/10.32614/CRAN.package.epiR

**Annex I**


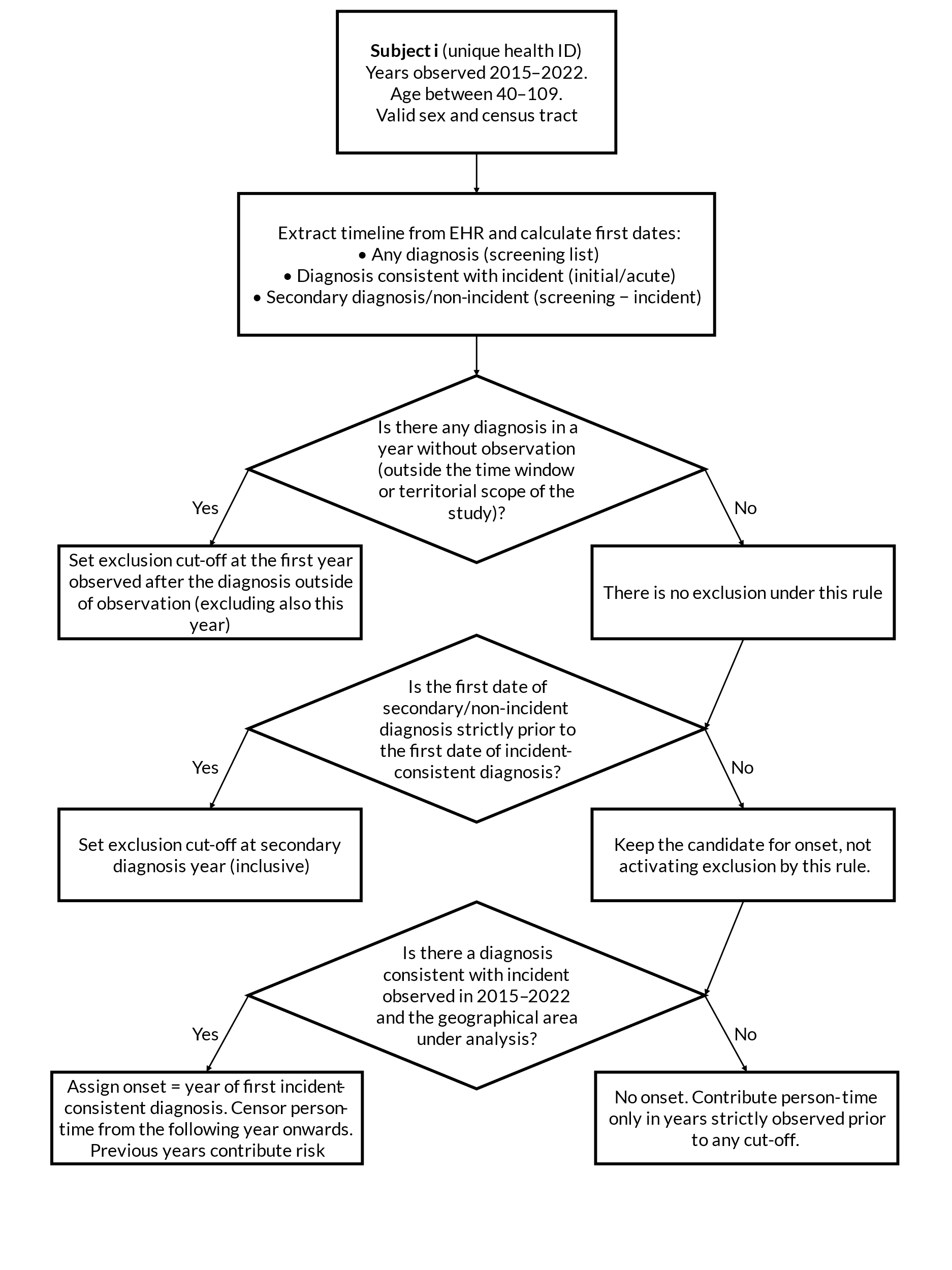


**Figure S.A.1**. Subject-level algorithm for retrospective cohort construction, exclusion cut-offs and incident onset assignment (2015–2022).

**Observation frame**. Individuals contribute person-years only within 2015-2022 and only for calendar years in which they have a non-missing census tract identifier inside the study area. “Under observation” includes both first entry and any later (re-)entries to the study area.

**Regional morbidity archive**. EHRs for the area defined in Figure S1 have been available since the 1990s and are searched longitudinally to detect prior diagnoses, even if recorded outside the study area or before 2015. This long look-back reduces left-censoring of prevalent disease.

**Prevalent outside observation**. If any screening-set code is recorded in a year when the person is not under observation, the individual is treated as prevalent at the first subsequent (re-)entry year. The cut-off is inclusive: that year contributes no at-risk person-time.

**Secondary before primary**. If the earliest secondary/non-incident diagnosis precedes the earliest incident-compatible diagnosis, exclusion starts from that calendar year onwards (inclusive), assuming pre-existing disease.

**Within-year ordering**. When multiple diagnoses occur in the same calendar year, precedence is determined by event dates. A secondary diagnosis recorded strictly before a primary one triggers exclusion from that calendar year onwards. A secondary recorded after a primary diagnosis occurs post-onset and does not alter incident status. When a primary and a secondary code share the exact same event date, the episode is classified as incident-compatible and does not trigger secondary-based exclusion.

**Incident onset**. An incident case is assigned only if the earliest incident-compatible diagnosis occurs during 2015-2022 while the person is under observation and, by construction, before any inclusive cut-off. Each person has at most one onset year.

**Censoring and denominators**. Person-time is accrued only in observed years strictly prior to any inclusive cut-off. From the onset year onward, no further at-risk time is accrued. Pre-onset observed years contribute to incidence denominators.

**Geographic and temporal limits**. Because valid census tract identifiers are only available from 2015, person-time is restricted to 2015-2022. Moves across municipal boundaries within a year and delayed coding may still introduce minor misclassification, partly mitigated by the regional EHR look-back.
