# Supplementary figures and images for: The spatial distribution of diagnosed Type 2 diabetes mellitus and cardiovascular disease incidence in Valencia, 2015–2022: a retrospective, registry-based study
Source: BMC Public Health. 2026 Mar 29;26:1495. doi: 10.1186/s12889-026-27160-3 (PMC13151391; doi:10.1186/s12889-026-27160-3)

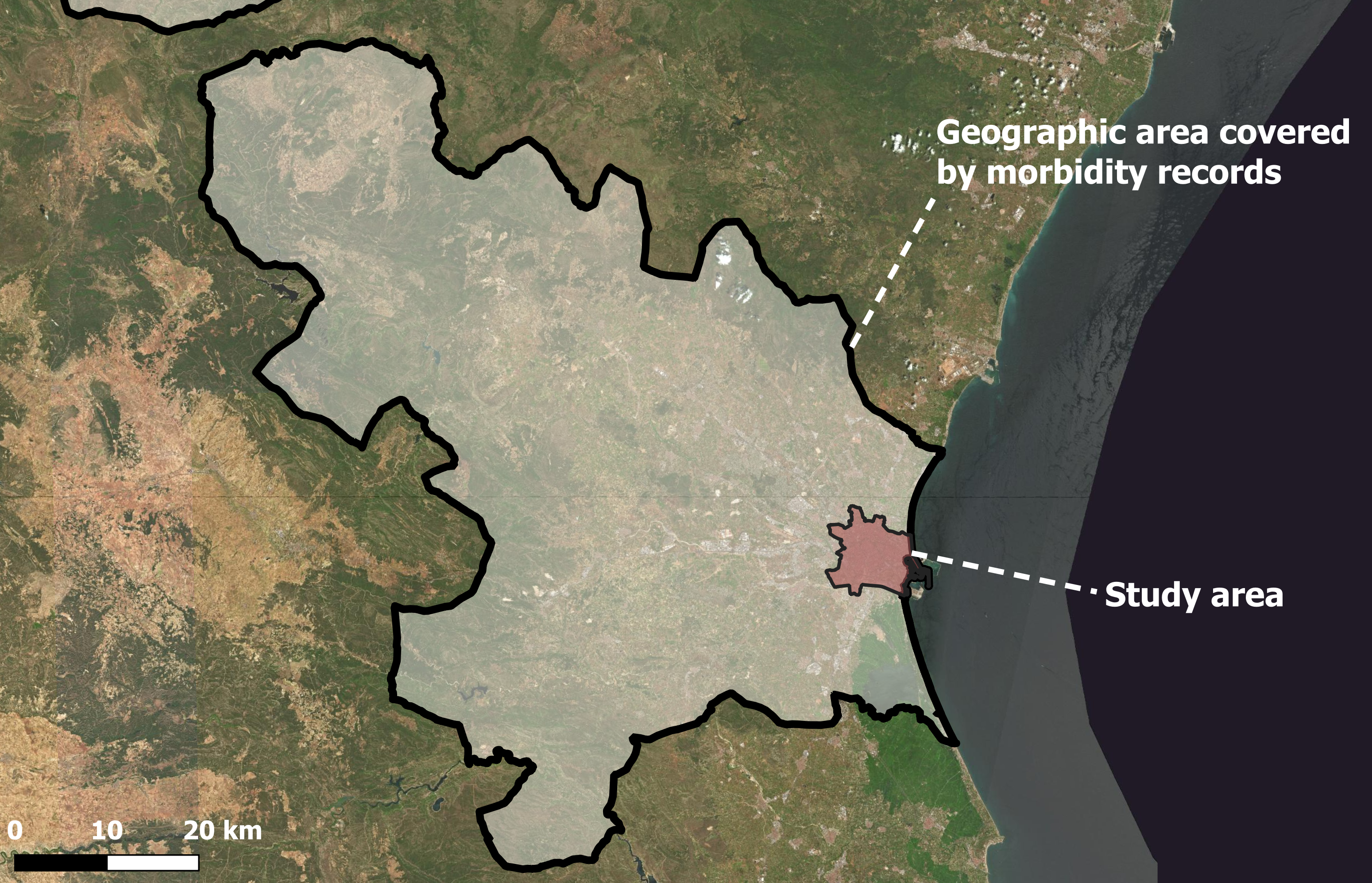

Supplement: Supplementary file 1 — Supplementary Material 1. [file 12889_2026_27160_MOESM1_ESM.zip › Figures/Figure S1.png]

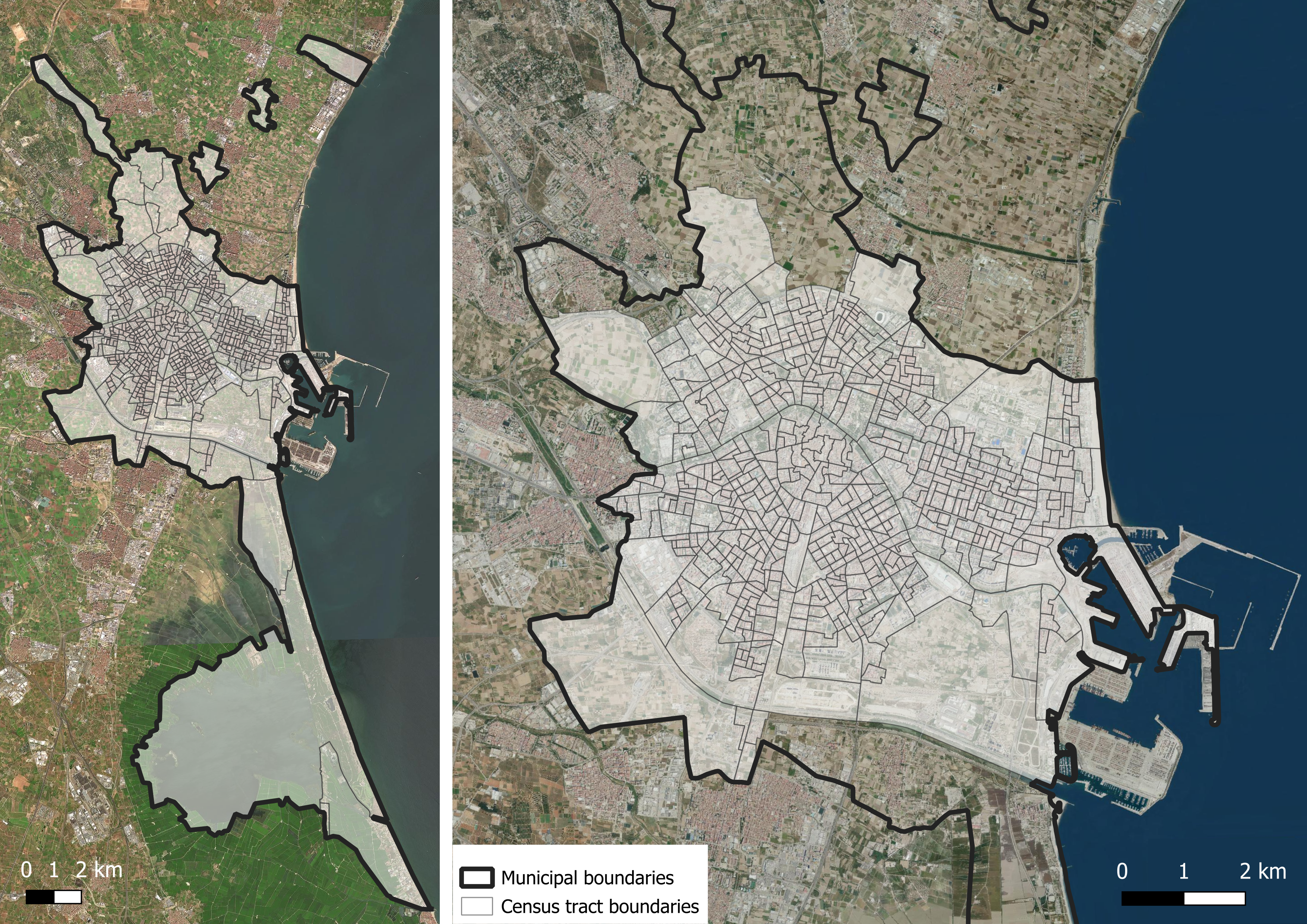

Supplement: Supplementary file 1 — Supplementary Material 1. [file 12889_2026_27160_MOESM1_ESM.zip › Figures/Figure S2.png]

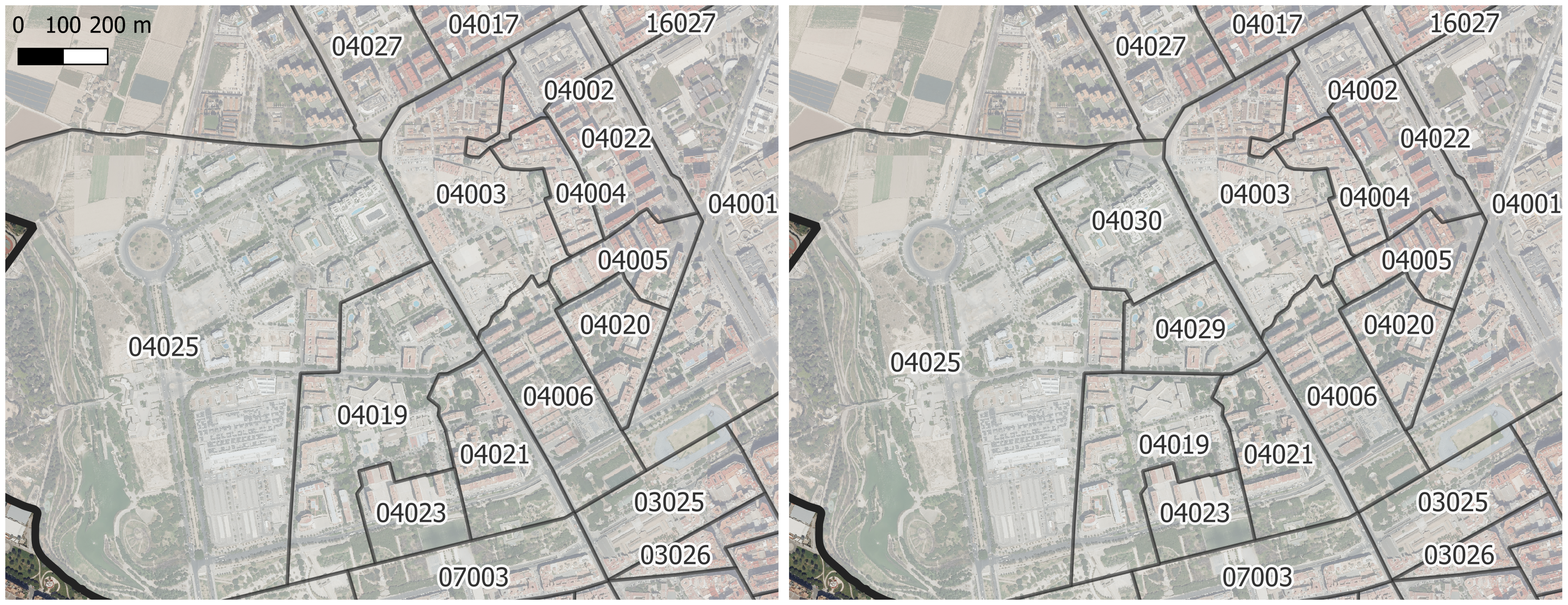

Supplement: Supplementary file 1 — Supplementary Material 1. [file 12889_2026_27160_MOESM1_ESM.zip › Figures/Figure S3.png]

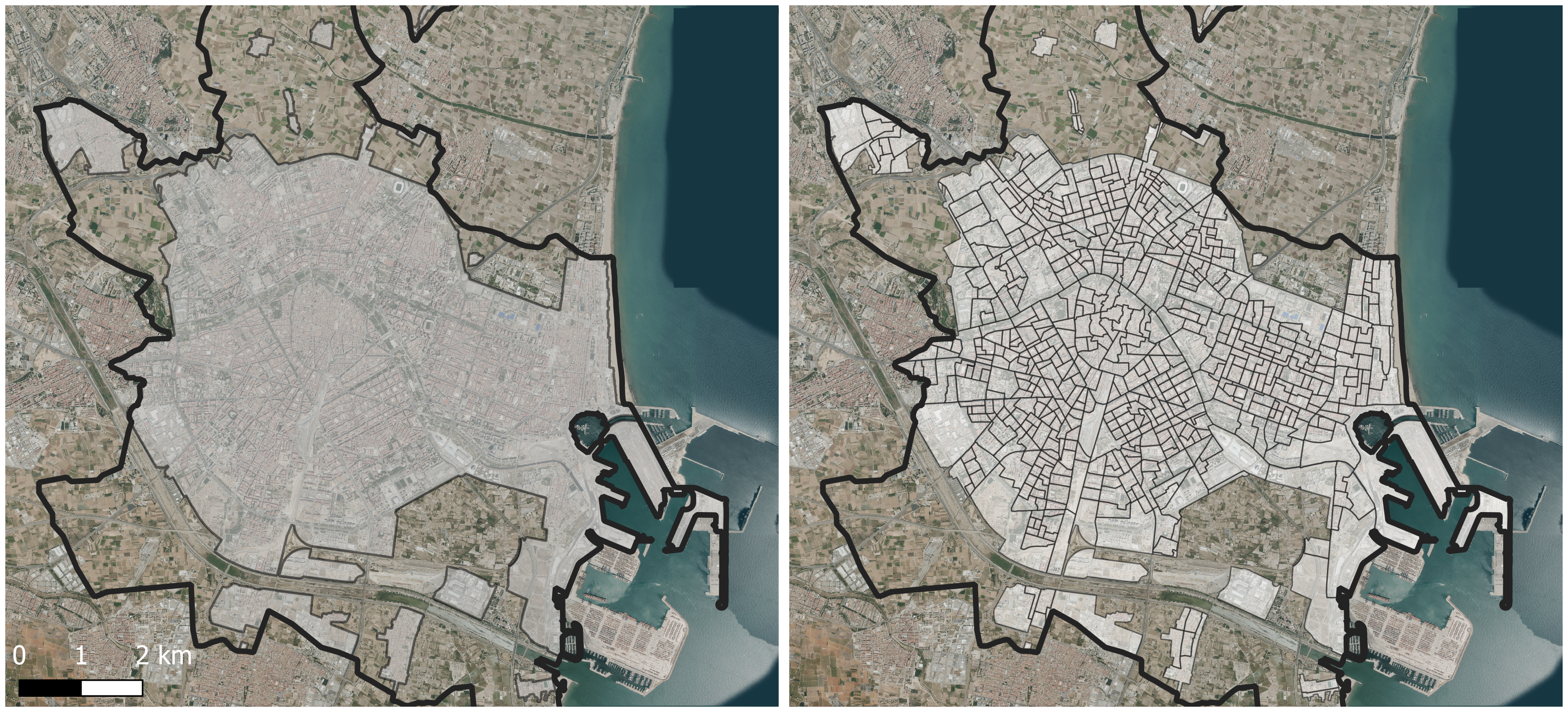

Supplement: Supplementary file 1 — Supplementary Material 1. [file 12889_2026_27160_MOESM1_ESM.zip › Figures/Figure S4.png]
